# Supplementary material for: Spectropolarimetric Holographic Multiplexing Metasurface With Super‐High Capacity Empowered by Mechanical Rotation
Source: Nanophotonics. 2026 May 5;15(9):e70117. doi: 10.1002/nap2.70117 (PMC13642513; doi:10.1002/nap2.70117)
Supplement: Supplementary file 1 [file NAP2-15-e70117-s003.pdf]

Supporting Information

Spectro-Polarimetric Holographic Multiplexing Metasurface with Super-High Capacity Empowered by Mechanical Rotation

*Ting Ma[1], Xianjin Liu[1], Qiwen Bao[1], Bolun Zhang[1] and Jun-Jun Xiao[1]**

[1]College of Integrated Circuits and Shenzhen Engineering Laboratory of Aerospace Detection and Imaging, Harbin Institute of Technology (Shenzhen), Shenzhen 518055, China

E-mail: eiexiao@hit.edu.cn

Contents

Supporting Note S1. Overview of metasurface multiplexing strategies 2
Supporting Note S2. Establishment of a surrogate model 2
Supporting Note S3. Jones matrix of a single meta-atom 4
A.Dispersive full-parameter Jones matrix of rectangular meta-atoms 4
B. Output field by metasurface for incident light at various wavelengths and polarization states 5
Supporting Note S4. Jones matrix of meta-atoms in a double-layer twist metasurface 7
A. Derivation of dispersive full-parameter Jones matrix about the overall rotation of the metasurface (meta-disk rotation) 7
B. Output field of twist metasurface with incident light at various wavelengths and polarization states 9
Supporting Note S5. Angular spectrum method 10
Supporting Note S6. Gradient backpropagation for end-to-end inverse design 11
Supporting Note S7. Evaluation metrics for holographic image quality 12
Supporting Note S8. Metasurface for wavelength, polarization, and diffraction distance multiplexing holography 13
A.Meta-diff-DONN for high-resolution image end-to-end inverse design 13
B. FDTD validation of the proposed methodology on a small-scale model 14
Supporting Note S9. Implementation details of the metasurface for polarization and rotation angle multiplexing 16
A. Meta-diff-DONN for high-resolution image end-to-end inverse design 16
B. The upper limit of frame rate in holographic video 21
Supporting Note S10. Implementation details of metasurface for wavelength, polarization, and rotation angle multiplexing co-design 22
A. Meta-diff-DONN for high-resolution image end-to-end inverse design 22
B. FDTD validation of the proposed methodology on a small-scale model 27
Supporting Note S11. Assessment of the impact of experimental error on the metasurface performance 30

Supporting Note S1. Overview of metasurface multiplexing strategies

To provide a comprehensive background on system capacity expansion in metasurfaces, Figure S1 visually summarizes the predominant strategies reported in recent literature. As discussed in the main manuscript, a majority of current studies primarily focus on three aspects of multiplexing strategies : (1) multiplexing in the dimension of optical parameters (such as wavelength, polarization, transmission direction, and orbital angular momentum); (2) electrical domain reconfiguration (utilizing active components like spatial light modulators or phase-change materials); and (3) mechanical reconfiguration (involving operations such as translation, rotation, or permutation). This brief literature overview serves as a broader context for the evolution of high-capacity holographic storage. It highlights the inherent trade-offs between channel capacity and imaging fidelity, thereby outlining the motivation for our proposed mechano-optical hybrid reconstruction framework detailed in the main text.

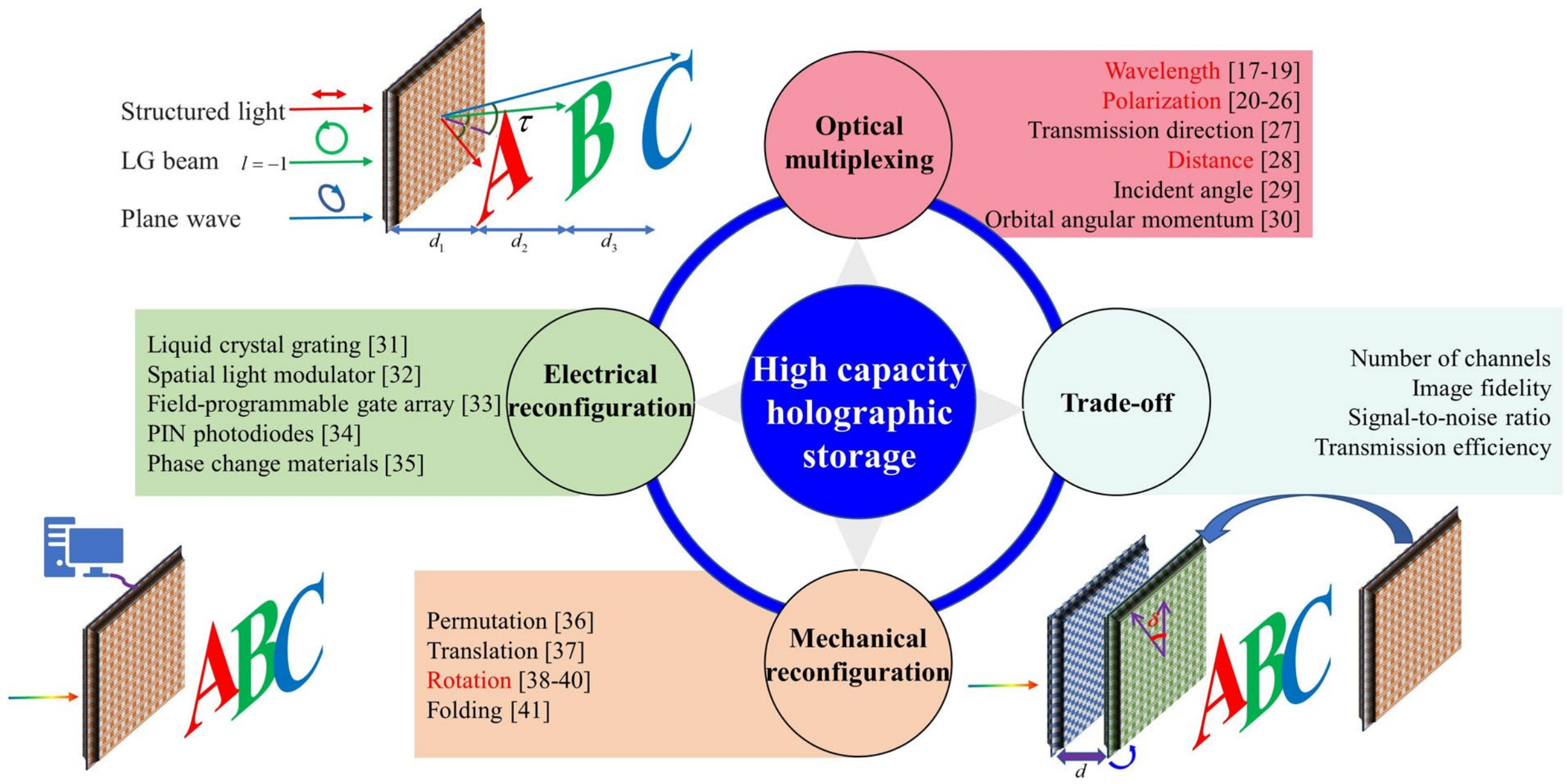

Figure S1. Three methods for expanding the storage capacity of metasurfaces as reported in the literature: optical parameter multiplexing, electrical domain reconfiguration, and mechanical reconfiguration. This work employs a combination of mechanical reconfiguration and optical parameter multiplexing, specifically highlighted in red. LG beam: Laguerre-Gaussian beam. The parameter l represents the topological charge of the OAM state. The parameter τ denotes the spatial diffraction angle between different multiplexed holographic channels. Note: The reference numbers cited in this figure correspond to the reference list in the main manuscript.

Supporting Note S2. Establishment of a surrogate model

To investigate the optical response characteristics of rectangular meta-atoms under different wavelengths and polarization states, we utilized the rigorous coupled wave analysis (RCWA) method, implemented using the TensorFlow framework version 2.13.1, to conduct a parameter scan on

rectangular meta-atoms with the following setup: Under illumination by RGB color and *x*-polarized incident light propagating along the negative *z*-axis. The period of the meta-atom was set to *P*=400 nm, with a fixed height of *H*=600 nm. The substrate material is silica (SiO_2), which has a constant refractive index of approximately 1.46 in the visible wavelength range. According to the equivalent refractive index theory, the greater the refractive index difference between the substrate and the nanoblock, the more likely it is to generate transmission phases covering the 0-2π range. Therefore, titanium dioxide (TiO_2 with a refractive index ranging from 2.5 to 2.85 in the visible band) was chosen as the dielectric material for the meta-atom. Considering practical fabrication limitations, the minimum size of the meta-atom was set to 50 nm. We sampled 101 length (*L*) and width (*W*) of the meta-atoms within the range of 50-250 nm at equal intervals of 2 nm, and selected three discrete working wavelengths $\lambda_1 = 450$ nm, $\lambda_2 = 532$ nm, $\lambda_3 = 635$ nm to collect the training data.

We calculated the corresponding transmission coefficient (both amplitude and phase) of these meta-atoms under *x*-axis linear polarization (*x*-pol) plane wave illumination at the three wavelengths, resulting in a database containing 30,603 samples. By symmetry, the transmission coefficient for *y*-axis linear polarization (*y*-pol) light can be obtained by transposing the results of *x*-pol. To achieve differentiable mapping of the meta-atoms transmission coefficients concerning geometric parameters, we construct a deep neural network (DNN) surrogate model to fit this complex mapping relationship. The reliability of the meta-atom transmission coefficients calculated through this DNN surrogate model was verified. Figure 2(b) shows the comparison of various geometric parameters at the three particular wavelengths, respectively obtained using the RCWA and DNN.

Figure S2(a) shows the structure of the DNN consisting of an input layer, three hidden layers (256-256-256 neurons), and an output layer, with ReLU serving as the activation function. The input layer receives the wavelength of the incident light and the geometric parameters (L, W) of the rectangular meta-atoms. The output layer encodes the amplitude (A_x) and phase (φ_x) of the output light field

$$t_x = A_x \mathrm{e}^{\mathrm{j}\varphi_x} \tag{S1}$$

We have used the dataset obtained from RCWA simulations to train and test the DNN, with a splitting ratio of 8:2. The gradient descent algorithm was employed to minimize the mean squared error (MSE), using the Adam optimizer in PyTorch 1.10.1 with a learning rate of 0.001 over 100,000 epochs. The loss function reads

$$\arg\min_{w} \frac{1}{N} \sum_{\lambda_i} (\mathbf{O} - g_{NN}(\mathbf{I}))^2 \tag{S2}$$

where w represents the weight parameters of the three fully connected neural network layers, $\lambda_i (i = 1,2,3; \lambda_i \in [450\text{ nm}, 532\text{ nm}, 635\text{ nm}])$ is the sampled wavelength, $\mathbf{I} = \{\lambda, L, W\}$ and $\mathbf{O} = \{A_x, \varphi_x\}$ denote the input and output of the neural network, respectively. Figure S2(b) shows the convergence curves during the training and the validation, with minimal differences between them, indicating that the DNN is well-trained. By swapping the length and width of the meta-atoms, the exact DNN model trained on the x-polarized light meta-atom library can be used to predict the optical response of y-polarized light on the meta-atoms.

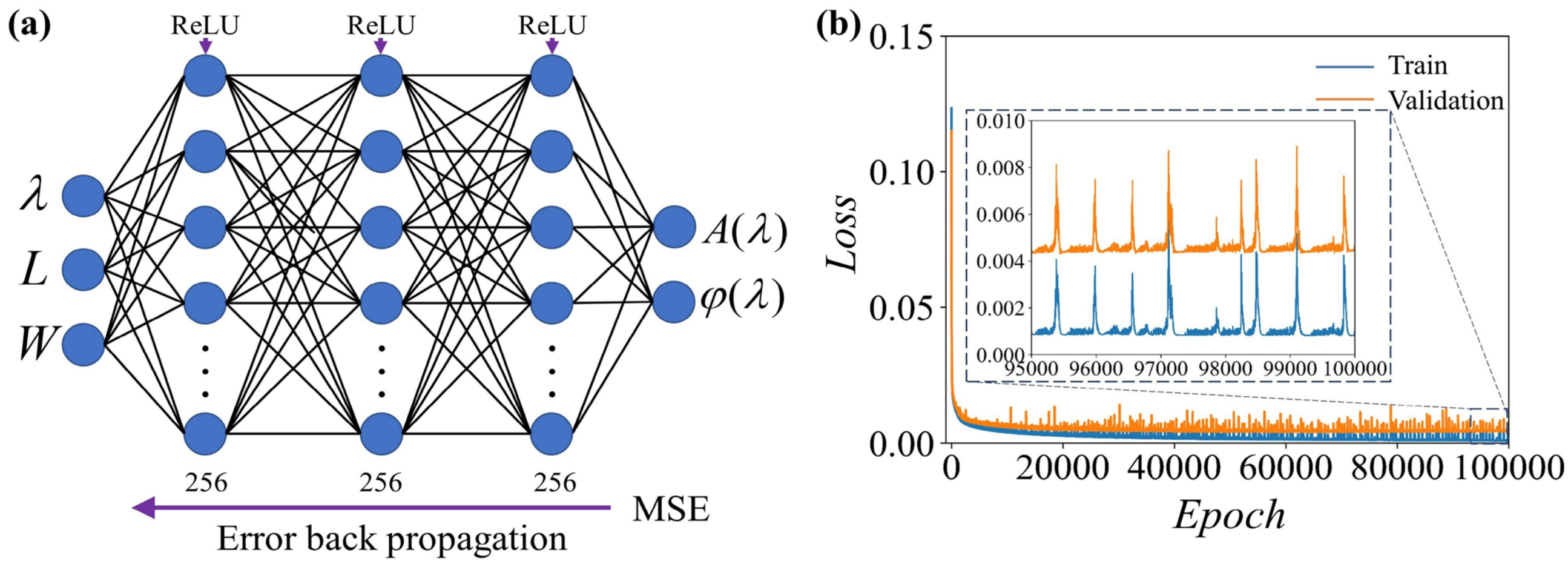

Figure S2. Structure and training of the DNN for meta-atom transmission coefficient modeling. (a) Structure of the DNN. (b)The loss convergence curve of the DNN train and validation processes, based on the x-pol light meta-atom data obtained by RCWA.

Supporting Note S3. Jones matrix of a single meta-atom

A. Dispersive full-parameter Jones matrix of rectangular meta-atoms

The metasurface consists of a two-dimensional array of nanoblock meta-atoms with a height of $H = 600\,\text{nm}$ as the basic unit, characterized by three variable geometric parameters: length (L), width (W), and orientation angle (θ). The optical responses of these engineered meta-atoms at each spatial coordinate (x, y) across the metasurface are locally modeled through a Jones matrix formalism that effectively describes a conventional linear birefringent waveplate:

$$
\begin{aligned}
M(\lambda) &= R(\theta)M_0(\lambda,\sigma;L,W)R(-\theta) \\
&= R(\theta)\begin{bmatrix} t_u(\lambda) & 0 \\ 0 & t_v(\lambda) \end{bmatrix} R(-\theta) \\
&= \begin{bmatrix} t_u(\lambda)\cos^2\theta + t_v(\lambda)\sin^2\theta & (t_u(\lambda)-t_v(\lambda))\sin\theta\cos\theta \\ (t_u(\lambda)-t_v(\lambda))\sin\theta\cos\theta & t_u(\lambda)\sin^2\theta + t_v(\lambda)\cos^2\theta \end{bmatrix} \\
&= \begin{bmatrix} t_{xx} & t_{xy} \\ t_{yx} & t_{yy} \end{bmatrix}
\end{aligned} \tag{S3}
$$

where the rotation matrix is $R(\theta)=\begin{bmatrix} \cos\theta & -\sin\theta \\ \sin\theta & \cos\theta \end{bmatrix}$. When the local meta-atom counterclockwise rotates, θ is positive. Note that $M_0(\lambda,\sigma;L,W\,|\,(x,y))$ is the Jones matrix when the orientation angle of the meta-atom is $\theta=0$, which is determined by its length (L), width (W), and the incident light's wavelength (λ) and polarization state (σ). Its two orthogonal components $t_u(\lambda)$ and $t_v(\lambda)$ represent the long axis and the short axis responses, which are both wavelength-dependent, offering the flexibility for wavelength multiplexing. In our case $t_{yx}(\lambda)=t_{xy}(\lambda)$, therefore, the metasurface composed of a single nanoblock inside a unit cell has at most 3 independent polarization channels.

B. Output field by metasurface for incident light at various wavelengths and polarization states

The incident light with an arbitrary polarization state can be described as $U_{in}=\begin{bmatrix} \cos\omega \\ e^{i\phi}\sin\omega \end{bmatrix}$, where ω represents the angle between the polarization direction of the light and the horizontal direction, and ϕ represents the phase shift of the light in the x-direction and y-direction. We use linearly polarized light for incidence so $\phi=0$ and $U_{\text{in}}=\begin{bmatrix} \cos\omega \\ \sin\omega \end{bmatrix}$. Therefore, with different orientations of the meta-atoms, there is a pixel-wise polarization conversion from incident light to transmitted light.

The light fields under normal incidence transmitted through the first metasurface are then given by

$$\begin{aligned} U_{\text{out}} &= M(\lambda)U_{\text{in}} \\ &= \begin{bmatrix} t_u(\lambda)\cos^2\theta + t_v(\lambda)\sin^2\theta & (t_u(\lambda)-t_v(\lambda))\sin\theta\cos\theta \\ (t_u(\lambda)-t_v(\lambda)\sin\theta\cos\theta & t_u(\lambda)\sin^2\theta + t_v(\lambda)\cos^2\theta \end{bmatrix}\begin{bmatrix}\cos\omega \\ \sin\omega\end{bmatrix} \\ &= \begin{bmatrix} (t_u(\lambda)\cos^2\theta + t_v(\lambda)\sin^2\theta)\cos\omega + (t_u(\lambda)-t_v(\lambda))\sin\theta\cos\theta\sin\omega \\ (t_u(\lambda)-t_v(\lambda))\sin\theta\cos\theta\cos\omega + (t_u(\lambda)\sin^2\theta + t_v(\lambda)\cos^2\theta)\sin\omega \end{bmatrix} \end{aligned} \tag{S4}$$

The x and y linear polarized light input and output $(x \to x, x \to y, y \to x, y \to y)$ by the dispersion full-parameter Jones matrix yield the following combinations:

$$\begin{aligned} U_{out}^{xx}(\lambda) &= \begin{bmatrix}1 & 0\\0 & 0\end{bmatrix}\begin{bmatrix} t_u(\lambda)\cos^2\theta + t_v(\lambda)\sin^2\theta & (t_u(\lambda)-t_v(\lambda))\sin\theta\cos\theta \\ (t_u(\lambda)-t_v(\lambda))\sin\theta\cos\theta & t_u(\lambda)\sin^2\theta + t_v(\lambda)\cos^2\theta \end{bmatrix}\begin{bmatrix}1\\0\end{bmatrix} \\ &= \begin{bmatrix} t_u(\lambda)\cos^2\theta + t_v(\lambda)\sin^2\theta \\ 0 \end{bmatrix} \end{aligned} \tag{S5}$$

$$\begin{aligned} U_{out}^{xy}(\lambda) &= \begin{bmatrix}0 & 0\\0 & 1\end{bmatrix}\begin{bmatrix} t_u(\lambda)\cos^2\theta + t_v(\lambda)\sin^2\theta & (t_u(\lambda)-t_v(\lambda))\sin\theta\cos\theta \\ (t_u(\lambda)-t_v(\lambda))\sin\theta\cos\theta & t_u(\lambda)\sin^2\theta + t_v(\lambda)\cos^2\theta \end{bmatrix}\begin{bmatrix}1\\0\end{bmatrix} \\ &= \begin{bmatrix} 0 \\ (t_u(\lambda)-t_v(\lambda))\sin\theta\cos\theta \end{bmatrix} \end{aligned} \tag{S6}$$

$$\begin{aligned} U_{out}^{yx}(\lambda) &= \begin{bmatrix}1 & 0\\0 & 0\end{bmatrix}\begin{bmatrix} t_u(\lambda)\cos^2\theta + t_v(\lambda)\sin^2\theta & (t_u(\lambda)-t_v(\lambda))\sin\theta\cos\theta \\ (t_u(\lambda)-t_v(\lambda))\sin\theta\cos\theta & t_u(\lambda)\sin^2\theta + t_v(\lambda)\cos^2\theta \end{bmatrix}\begin{bmatrix}0\\1\end{bmatrix} \\ &= \begin{bmatrix} (t_u(\lambda)-t_v(\lambda))\sin\theta\cos\theta \\ 0 \end{bmatrix} \end{aligned} \tag{S7}$$

$$\begin{aligned} U_{out}^{yy}(\lambda) &= \begin{bmatrix}0 & 0\\0 & 1\end{bmatrix}\begin{bmatrix} t_u(\lambda)\cos^2\theta + t_v(\lambda)\sin^2\theta & (t_u(\lambda)-t_v(\lambda))\sin\theta\cos\theta \\ (t_u(\lambda)-t_v(\lambda))\sin\theta\cos\theta & t_u(\lambda)\sin^2\theta + t_v(\lambda)\cos^2\theta \end{bmatrix}\begin{bmatrix}0\\1\end{bmatrix} \\ &= \begin{bmatrix} 0 \\ t_u(\lambda)\sin^2\theta + t_v(\lambda)\cos^2\theta \end{bmatrix} \end{aligned} \tag{S8}$$

According to $I = |U_x|^2 + |U_y|^2$, it's obvious that $\left|I_{out}^{xy}(\lambda)\right| = \left|I_{out}^{yx}(\lambda)\right|$. Therefore, there are 3 independent polarization conversion channels $(x \to x, x \to y, y \to y)$. This is the fundamental limitation of the polarization multiplexing capability of a single-layer metasurface, which stems from the dimensionality constraint of the Jones matrix.

Supporting Note S4. Jones matrix of meta-atoms in a double-layer twist metasurface

A. Derivation of dispersive full-parameter Jones matrix about the overall rotation of the metasurface (meta-disk rotation)

We keep the first metasurface stationary, and rotate the second metasurface counterclockwise by an angle δ, causing a relative twist in the diffraction domain between the two metasurfaces. The Jones matrix of the local meta-atoms on the first metasurface is consistent with the derivation result in Supporting Note S3. As shown in Figure S3(a), the overall rotation of the second metasurface will cause changes in the position coordinates and orientation angles of each local meta-atom:

$$\begin{cases} \begin{bmatrix} \tilde{x} \\ \tilde{y} \end{bmatrix} = R_{global}(\delta) \begin{bmatrix} x \\ y \end{bmatrix} \\ \psi = \theta_2 + \delta \end{cases} \tag{S9}$$

The rotation angle $(\delta > 0)$ of the metasurface is positive in the counterclockwise direction. The overall rotation matrix of the metasurface implies meta-atom coordinate transformation via $R_{global}(\delta) = \begin{bmatrix} \cos(\delta) & -\sin(\delta) \\ \sin(\delta) & \cos(\delta) \end{bmatrix}$, where (x, y) is the original coordinate of the meta-atom and $(\tilde{x}, \tilde{y})$ is the coordinate after rotation. θ_2 is the original orientation angle of the local meta-atom on the second metasurface, and ψ is the orientation angle after the overall rotation of the metasurface.

Represent this process in the form of a Jones matrix:

$$\begin{aligned} \tilde{M}_2(\lambda; \mathbf{G}_2) &= R(\psi) M_{2,0}(\lambda, \sigma; \mathbf{G}_2) R(-\psi) \\ &= R(\psi) \begin{bmatrix} t_u(\lambda) & 0 \\ 0 & t_v(\lambda) \end{bmatrix} R(-\psi) \\ &= \begin{bmatrix} t_u(\lambda)\cos^2\psi + t_v(\lambda)\sin^2\psi & (t_u(\lambda) - t_v(\lambda))\sin\psi\cos\psi \\ (t_u(\lambda) - t_v(\lambda))\sin\psi\cos\psi & t_u(\lambda)\sin^2\psi + t_v(\lambda)\cos^2\psi \end{bmatrix} \end{aligned} \tag{S10}$$

We have made a short notation $\mathbf{G}_2$ for the combination $\{L_2, W_2, \theta_2\}$. The new coordinates after the meta-disk rotation are

$$\begin{cases} \tilde{x} = x \cdot \cos(\delta) - y \cdot \sin(\delta) \\ \tilde{y} = x \cdot \sin(\delta) + y \cdot \cos(\delta) \end{cases} \tag{S11}$$

Figure S3(b) describes two interpolation methods for the new coordinates in the meta-disk grid.

(1) Using Nearest-neighbor interpolation, the transmission coefficient of the meta-atom is filled into the nearest neighbor coordinate position.

$$t_{nearest}(x,y) = h(\lfloor \tilde{x} \rfloor, \lfloor \tilde{y} \rfloor) \tag{S12}$$

Here, $\lfloor \tilde{x} \rfloor$ and $\lfloor \tilde{y} \rfloor$ respectively represent the nearest integer values of $\tilde{x}$ and $\tilde{y}$, h is the mapping function of the transmission coefficient of the meta-atom at the position coordinates (x,y) after rotation.

(2) Using the bilinear interpolation, calculate the weighted average of the transmission coefficients of the four meta-atoms around the target position, and set it to the meta-atom at the target position.

$$\begin{aligned} t_{bilinear}(x,y) = {} & (1-\alpha)(1-\beta)h(\lfloor \tilde{x} \rfloor, \lfloor \tilde{y} \rfloor) + \alpha(1-\beta)h(\lfloor \tilde{x} \rfloor + 1, \lfloor \tilde{y} \rfloor) \\ & + (1-\alpha)\beta h(\lfloor \tilde{x} \rfloor, \lfloor \tilde{y} \rfloor + 1) + \alpha\beta h(\lfloor \tilde{x} \rfloor + 1, \lfloor \tilde{y} \rfloor + 1) \end{aligned} \tag{S13}$$

Here, $\alpha = \tilde{x} - \lfloor \tilde{x} \rfloor$ and $\beta = \tilde{y} - \lfloor \tilde{y} \rfloor$ respectively represent the horizontal and vertical offsets of the target point in the rotated image. Bilinear interpolation utilizes more information from the meta-atoms, making the calculation of the modulated light field after rotation more accurate. We have utilized this in our design.

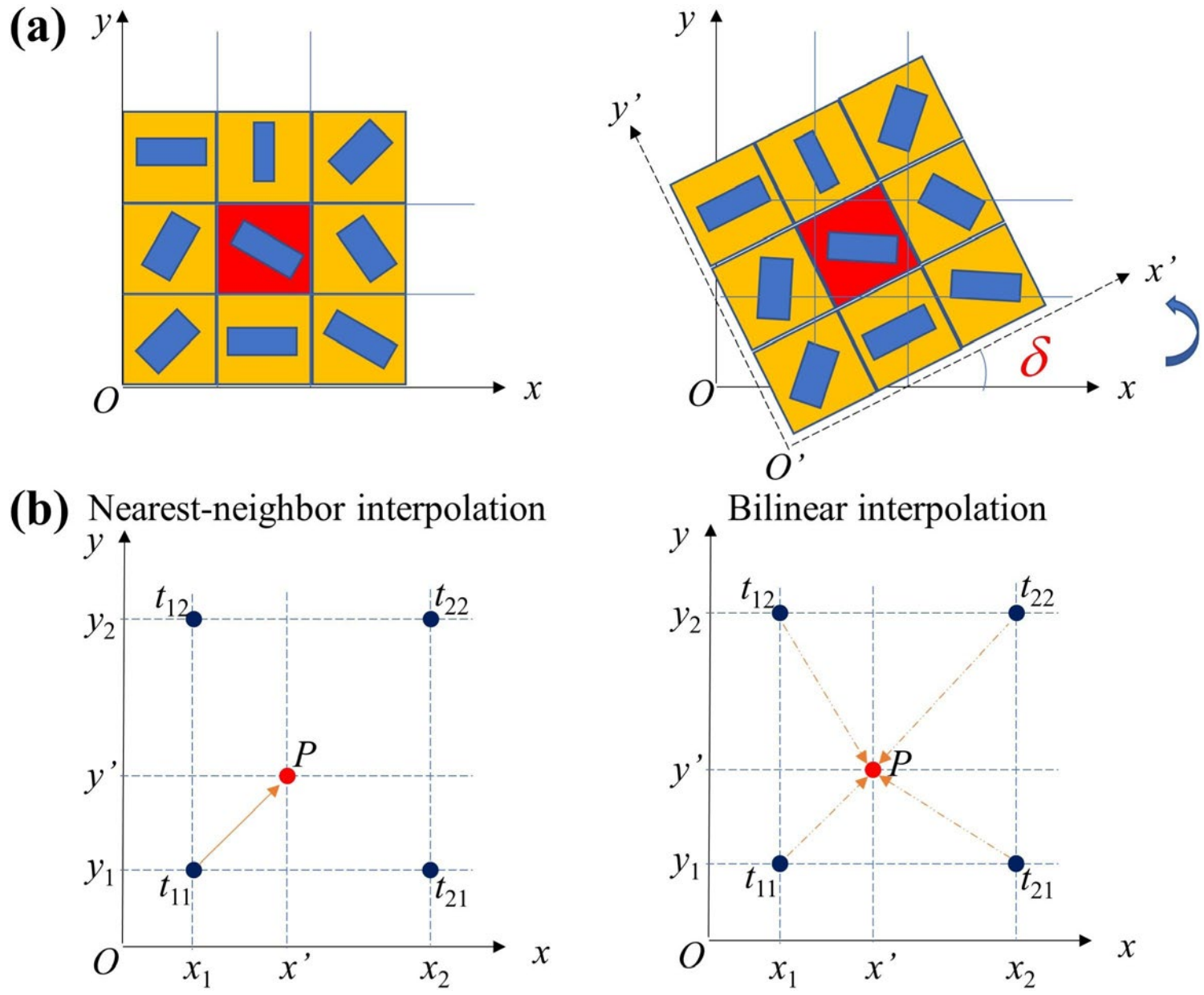

Figure S3. Schematic diagram of the metasurface overall rotation and interpolation algorithm for defining the meta-atom's new position. (a) The change of local meta-atom orientation and position caused by the metasurface's overall rotation. (b) Schematic of nearest-neighbor interpolation and the bilinear interpolation.

B. Output field of twist metasurface with incident light at various wavelengths and polarization states

The incident linearly polarized light can be described as $U_{\text{in}}=\begin{bmatrix}\cos\omega\\ \sin\omega\end{bmatrix}$. Therefore, with different orientations of the meta-atoms, there is a pixel-wise polarization conversion from incident light to transmitted light.

According to Equations (S4) and (S10), the output light fields under normal incidence transmitted through the first metasurface are then given by

$$U_{\text{out}}=M_2(\lambda)M_1(\lambda)U_{\text{in}}$$
$$=\begin{bmatrix}t_{u_2}(\lambda)\cos^2\psi+t_{v_2}(\lambda)\sin^2\psi & (t_{u_2}(\lambda)-t_{v_2}(\lambda))\sin\psi\cos\psi\\ (t_{u_2}(\lambda)-t_{v_2}(\lambda))\sin\psi\cos\psi & t_{u_2}(\lambda)\sin^2\psi+t_{v_2}(\lambda)\cos^2\psi\end{bmatrix}\begin{bmatrix}t_{u_1}(\lambda)\cos^2\theta_1+t_{v_1}(\lambda)\sin^2\theta_1 & (t_{u_1}(\lambda)-t_{v_1}(\lambda))\sin\theta_1\cos\theta_1\\ (t_{u_1}(\lambda)-t_{v_1}(\lambda))\sin\theta_1\cos\theta_1 & t_{u_1}(\lambda)\sin^2\theta_1+t_{v_1}(\lambda)\cos^2\theta_1\end{bmatrix}\begin{bmatrix}\cos\omega\\ \sin\omega\end{bmatrix} \tag{S14}$$

The x and y linear polarized light input and output $(x\rightarrow x, x\rightarrow y, y\rightarrow x, y\rightarrow y)$ by the dispersion full-parameter Jones matrix yield the following combinations:

$$U_{out}^{xx}(\lambda)=\begin{bmatrix}1&0\\0&0\end{bmatrix}M_2(\lambda)M_1(\lambda)\begin{bmatrix}1\\0\end{bmatrix}$$
$$=\begin{bmatrix}(t_{u_2}(\lambda)\cos^2\psi+t_{v_2}(\lambda)\sin^2\psi)(t_{u_1}(\lambda)\cos^2\theta_1+t_{v_1}(\lambda)\sin^2\theta_1)+(t_{u_2}(\lambda)-t_{v_2}(\lambda))\sin\psi\cos\psi(t_{u_1}(\lambda)-t_{v_1}(\lambda))\sin\theta_1\cos\theta_1\\ 0\end{bmatrix} \tag{S15}$$

$$U_{out}^{xy}(\lambda)=\begin{bmatrix}0&0\\0&1\end{bmatrix}M_2(\lambda)M_1(\lambda)\begin{bmatrix}1\\0\end{bmatrix}$$
$$=\begin{bmatrix}0\\ (t_{u_2}(\lambda)-t_{v_2}(\lambda))\sin\psi\cos\psi(t_{u_1}(\lambda)\cos^2\theta_1+t_{v_1}(\lambda)\sin^2\theta_1)+(t_{u_2}(\lambda)\sin^2\psi+t_{v_2}(\lambda)\cos^2\psi)(t_{u_1}(\lambda)-t_{v_1}(\lambda))\sin\theta_1\cos\theta_1\end{bmatrix} \tag{S16}$$

$$U_{out}^{yx}(\lambda)=\begin{bmatrix}1&0\\0&0\end{bmatrix}M_2(\lambda)M_1(\lambda)\begin{bmatrix}0\\1\end{bmatrix}$$
$$=\begin{bmatrix}(t_{u_2}(\lambda)\cos^2\psi+t_{v_2}(\lambda)\sin^2\psi)(t_{u_1}(\lambda)-t_{v_1}(\lambda))\sin\theta_1\cos\theta_1+(t_{u_2}(\lambda)-t_{v_2}(\lambda))\sin\psi\cos\psi(t_{u_1}(\lambda)\sin^2\theta_1+t_{v_1}(\lambda)\cos^2\theta_1)\\ 0\end{bmatrix} \tag{S17}$$

$$U_{out}^{yy}(\lambda)=\begin{bmatrix}0&0\\0&1\end{bmatrix}M_2(\lambda)M_1(\lambda)\begin{bmatrix}0\\1\end{bmatrix}$$
$$=\begin{bmatrix}0\\ (t_{u_2}(\lambda)-t_{v_2}(\lambda))\sin\psi\cos\psi(t_{u_1}(\lambda)-t_{v_1}(\lambda))\sin\theta_1\cos\theta_1+(t_{u_2}(\lambda)\sin^2\psi+t_{v_2}(\lambda)\cos^2\psi)(t_{u_1}(\lambda)\sin^2\theta_1+t_{v_1}(\lambda)\cos^2\theta_1)\end{bmatrix} \tag{S18}$$

Obviously, $I = |U_x|^2 + |U_y|^2$, so $\left|I_{out}^{xy}(\lambda)\right| \neq \left|I_{out}^{yx}(\lambda)\right|$, there are 4 independent polarization conversion channels $(x \to x, x \to y, y \to x, y \to y)$. Therefore, the dual-layer metasurface decouples the diagonal channels of the Jones matrix, and the dual-layer metasurface composed of individual nanoblocks possesses 4 independent polarization conversion channels.

Supporting Note S5. Angular spectrum method

In the optimized system, we simulate the propagation of the optical field using the angular spectrum method (ASM).

$$\begin{cases} U(x,y,z_{i+1}) = \mathcal{F}^{-1}\{\mathcal{F}\{U_0(x,y,z_i)\}\cdot H(f_x,f_y,z_{i+1})\} \\ H = \exp(\mathrm{j}kz\sqrt{1-(\lambda f_x)^2-(\lambda f_y)^2}), f_x^2+f_y^2 < \dfrac{1}{\lambda^2} \end{cases} \tag{S19}$$

where U_0 is the complex field propagates from plane z_i to plane z_{i+1}. Using the angular spectrum method, we obtain the input complex optical fields at Layer 1, Layer 2, and the sensor plane. Here, $\mathcal{F}\{\bullet\}$ and $\mathcal{F}^{-1}\{\bullet\}$ denote the Fourier transform and inverse Fourier transform, respectively. Complex amplitude modulation occurs via the Hadamard product of the incoming optical field and the transmission coefficient term. In this process, f_x and f_y represent the spatial frequencies in the x and y directions, z is the propagation distance. Finally, the sensor plane measures the intensity of the forward propagation output $I = \left|U(x,y,z_{i+1})\right|^2$. To prevent numerical spectral aliasing during FFT-based ASM calculations, zero-padding was applied to the optical field. This padding creates a spatial buffer zone to avoid wraparound artifacts and increases the frequency-domain sampling density ($\Delta f = 1/L$) to accurately resolve the rapid oscillations of the free-space transfer function over the propagation distance.

To clarify the comprehensive physical model describing the light evolution through the cascaded metasurface system, taking into account both local meta-atom modulation and free-space diffraction, we outline the step-by-step computational procedure as follows:

Step 1: Initialization and Layer-1 modulation. The incident plane wave $U_{in}(\lambda,\sigma)$ illuminates the first metasurface. The local transmitted field $U_1(\lambda,\sigma;x,y)$ is computed by multiplying the incident field by the dispersive full-parameter Jones matrix of the static meta-atoms $U_1(\lambda,\sigma;x,y) = M_1(\lambda,\sigma;x,y)\cdot U_{in}(\lambda,\sigma)$.

Step 2: First free-space propagation (field synthesis). The modulated field $U_1(\lambda,\sigma;x,y)$

propagates over the distance d to the front facet of the second metasurface. This propagation is rigorously computed using the ASM. Crucially, this step synthesizes the interference of all meta-atoms from the first layer, yielding the accurate intermediate incident field $U_{in,2}(\lambda,\sigma;x,y) = f_{propaga.}\langle U_1(\lambda,\sigma;x,y),d\rangle$.

Step 3: Dynamic coordinate alignment. As the second metasurface is mechanically rotated by an overall angle δ with respect to the optical axis, the spatial coordinates of its constituent meta-atoms change. Their new transmission coefficients are mapped onto the fixed computational grid utilizing bilinear interpolation, ensuring a differentiable and smooth representation of the rotated Jones matrix $M_2(\lambda,\sigma;x,y;\delta)$ (as detailed in Supporting Information Note S4).

Step 4: Layer-2 modulation. The synthetic intermediate field $U_{in,2}(\lambda,\sigma;x,y)$ is then modulated by the rotated second metasurface, yielding the transmitted field $U_2(\lambda,\sigma;x,y) = M_2(\lambda,\sigma;x,y;\delta)\cdot U_{in,2}(\lambda,\sigma;x,y)$.

Step 5: Second free-space propagation and detection. Finally, the output field $U_2(\lambda,\sigma;x,y;\delta)$ propagates over another distance z to the detector plane via the ASM, generating the final holographic complex amplitude $U_{out}(\lambda,\sigma;x,y) = f_{propaga.}\langle U_2(\lambda,\sigma;x,y;\delta),z\rangle$. The intensity of the forward propagation output, $I = |U_{out}|^2$, is then captured by the sensor plane.

Supporting Note S6. Gradient backpropagation for end-to-end inverse design

The output light field from the above model is used to define the loss function

$$Loss = \sum_{\lambda,\sigma,z} F[U_{out}(\lambda,\sigma,z;\mathbf{G}),U_{tar}(\lambda,\sigma,z)] \tag{S20}$$

Among them, $U_{out}(\lambda,\sigma,z;\mathbf{G})$ is the model output light field generated by the metasurface with structural parameters $\mathbf{G}$, and $U_{tar}(\lambda,\sigma,z)$ is the target light field expected to be output at the position (x,y,z). Here λ, σ and z represent the wavelength, polarization, and receiving plane distance, respectively. F is a function of the loss function.

The effect of the end-to-end model optimization is evaluated using the MSE and negative Pearson correlation coefficient (NPCC), which is defined as

$$MSE = \frac{1}{n}\sum_{i=1}^{n}(I_{out,i} - I_{tar,i})^2 \tag{S21}$$

$$NPCC = \frac{\sum_{i=1}^{n}(I_{out,i} - \overline{I_{out}})(I_{tar,i} - \overline{I_{tar}})}{\sqrt{\sum_{i=1}^{n}(I_{out,i} - \overline{I_{out}})^2}\sqrt{\sum_{i=1}^{n}(I_{tar,i} - \overline{I_{tar}})^2}} \tag{S22}$$

where $I_{out,i}$ and $I_{tar,i}$ are the i-th pixel units of the output light field intensity and the target light field intensity, respectively. n is the number of pixel units. $I_{out} = |U_{out}|^2$, $I_{tar} = |U_{tar}|^2$, $\overline{I_{out}}$ and $\overline{I_{tar}}$ are the average values of the output light field intensity and the target light field intensity, respectively. The loss function we use reads

$$Loss = a_1 MSE + a_2(1 - NPCC) \tag{S23}$$

Where a_1 and a_2 denote the balancing coefficients that regulate the trade-off between the MSE and NPCC in the loss function.

The forward simulator links the metasurface geometric layout $\mathbf{G}(L, W, \theta)$ with the diffraction modulation field U_M in a differentiable form, establishing this connection. This makes it possible to apply the chain rule through automatic differentiation. The gradient of the loss function to the optimizable variables is as follows：

$$\begin{cases} \frac{\partial Loss}{\partial L} = \frac{\partial Loss}{\partial U_{out}} \frac{\partial U_{out}}{\partial f_{propa.}} \frac{\partial f_{propa.}}{\partial M} \frac{\partial M}{\partial g_{NN}} \frac{\partial g_{NN}}{\partial L} \\ \frac{\partial Loss}{\partial W} = \frac{\partial Loss}{\partial U_{out}} \frac{\partial U_{out}}{\partial f_{propa.}} \frac{\partial f_{propa.}}{\partial M} \frac{\partial M}{\partial g_{NN}} \frac{\partial g_{NN}}{\partial W} \\ \frac{\partial Loss}{\partial \theta} = \frac{\partial Loss}{\partial U_{out}} \frac{\partial U_{out}}{\partial f_{propa.}} \frac{\partial f_{propa.}}{\partial M} \frac{\partial M}{\partial \theta} \end{cases} \tag{S24}$$

For simplicity, we refer to this chain-rule-based differentiable mode as a metasurface-based differentiable diffractive optical neural network (**meta-diff-DONN**).

Supporting Note S7. Evaluation metrics for holographic image quality

The performance of holographic reconstruction is evaluated using the structural similarity index measure (SSIM) and peak signal-to-noise ratio (PSNR). These metrics provide a comprehensive assessment of the quality of holographic reconstruction, capturing both pixel-level accuracy and perceptual similarity. The PSNR is the ratio of the energy of the peak signal to the average energy of

the noise. It is expressed in decibels (dB), with higher values indicating better reconstruction quality. MSE represents the mean squared error between the reconstructed holographic image and the target image. For two images I and T, their PSNR is defined

$$PSNR = 10\log_{10}\left(\frac{1}{MSE}\right) \tag{S25}$$

SSIM is a metric that measures the structural similarity between two images. It compares the images from three aspects: luminance, contrast, and structure, which align more closely with the human eye's judgment of image quality. For two images I and T, their SSIM is defined as

$$\begin{cases} SSIM(I,T) = [l(I,T)]^{\alpha}[c(I,T)]^{\beta}[s(I,T)]^{\gamma} \\ l(I,T) = \dfrac{2\mu_I\mu_T + C_1}{\mu_I^2 + \mu_T^2 + C_1} \\ c(I,T) = \dfrac{2\sigma_I\sigma_T + C_2}{\sigma_I^2 + \sigma_T^2 + C_2} \\ s(I,T) = \dfrac{\sigma_{IT} + C_3}{\sigma_I\sigma_T + C_3} \end{cases} \tag{S26}$$

Among them, $l(I,T)$ compares the brightness of two images, $c(I,T)$ compares the contrast, $s(I,T)$ compares the structure, α, β, γ are parameters that adjust the relative importance of the three. μ_I, μ_T are the average values of the two images, σ_I, σ_T the standard deviations of the two images, and σ_{IT} is the covariance of the two images, respectively. C_1, C_2, C_3 all of which are constants to keep the denominator from being zero. The range of SSIM is between -1 and 1, and the closer it is to 1, the higher the similarity between the two images.

For simplicity, the parameters $\alpha = \beta = \gamma = 1$, and $C_3 = C_2 / 2$, resulting in

$$SSIM(I,T) = \frac{(2\mu_I\mu_T + C_1)(2\sigma_{IT} + C_2)}{(\mu_I^2 + \mu_T^2 + C_1)(\sigma_I^2 + \sigma_T^2 + C_2)} \tag{S27}$$

Supporting Note S8. Metasurface for wavelength, polarization, and diffraction distance multiplexing holography

A. Meta-diff-DONN for high-resolution image end-to-end inverse design

In the end-to-end inverse design process, the geometric layout of the metasurface is directly optimized

starting from the target holographic image, greatly improving the optimization efficiency and eliminating any phase-matching errors. Our meta-diff-DONN model is implemented using PyTorch 1.10.1 on a workstation equipped with a Tesla V100 GPU (Nvidia Inc., 32GB VRAM) and an Intel Xeon Gold 5115 CPU (256GB RAM). To reduce numerical calculation errors, we improve the spectral sampling rate of the angular spectrum algorithm through zero-padding. In each training iteration, data pairs containing all channels are fed in batches into the training model, and the geometry $\{L, W, \theta\}$ of the metasurface is updated using the Adam optimizer with a learning rate of 0.01 for the backpropagation. The training process lasts approximately 2 hours, involving 2000 epochs to ensure sufficient optimization. Figure S4(a) displays a portion of the optimized metasurface geometric layout obtained from the end-to-end design, and the length, width, and orientation angle distribution of the optimized metasurface are given in the main text Figure 3(a). Figure S4(b) shows the loss curve of the dual-polarization color holographic end-to-end design. In the two-layer metasurface we designed, each layer includes 500×500 $(200\ \mu m \times 200\ \mu m)$ meta-atoms.

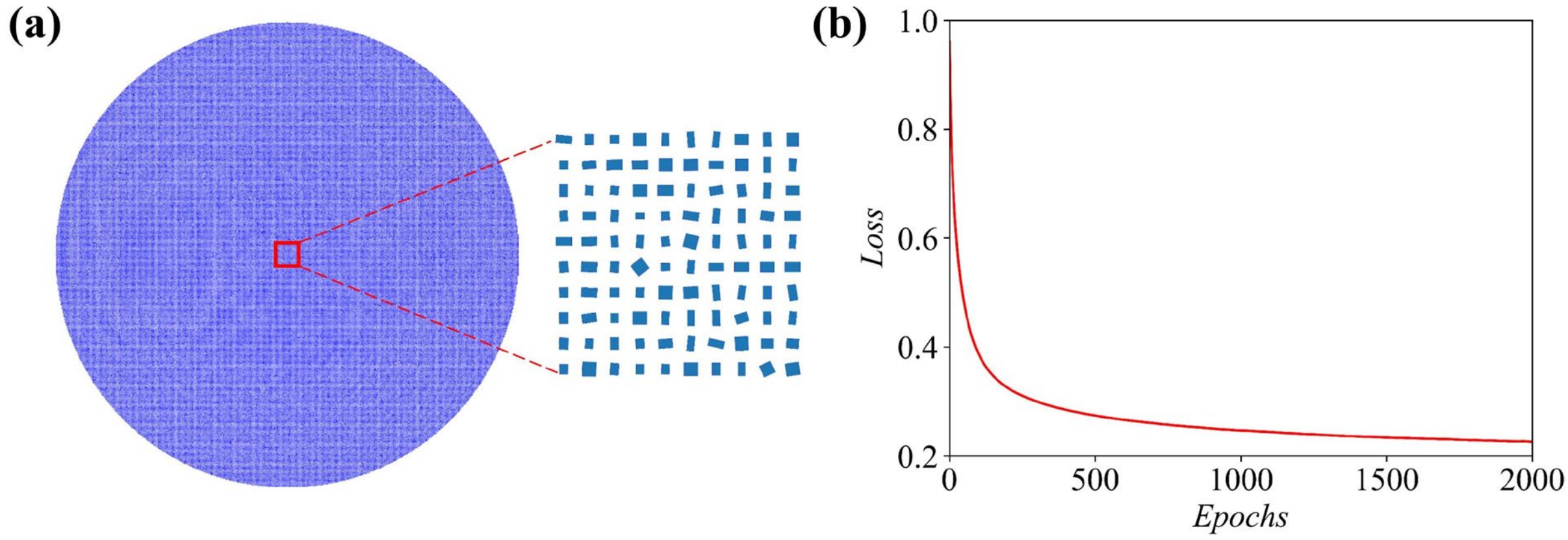

Figure S4. Wavelength, polarization, and diffraction distance multiplexing holography. (a) The optimized metasurface structure, with a zoom-in view showing the 245th to 255th units in both transverse and longitudinal directions. (b) Loss curve during optimization.

B. FDTD validation of the proposed methodology on a small-scale model

We validated the effectiveness of our proposed end-to-end design framework using FDTD full-wave simulations. Taking wavelength and polarization multiplexed holography as an example, due to limited computational resources, we utilized a metasurface with 100×100 pixels and set the diffraction distance $z = 60\ \mu m$. As shown in Figure S5(a), by switching the polarization states of the input and

output light, "RGB" and "HIT" color holograms can be obtained, respectively. Figure S5(b) illustrates the distribution maps of length, width, and orientation angle of the optimized metasurface based on the end-to-end design method. The geometric layout of the optimized metasurface is depicted in Figure S5(c). The loss curve during training is presented in Figure S5(d).

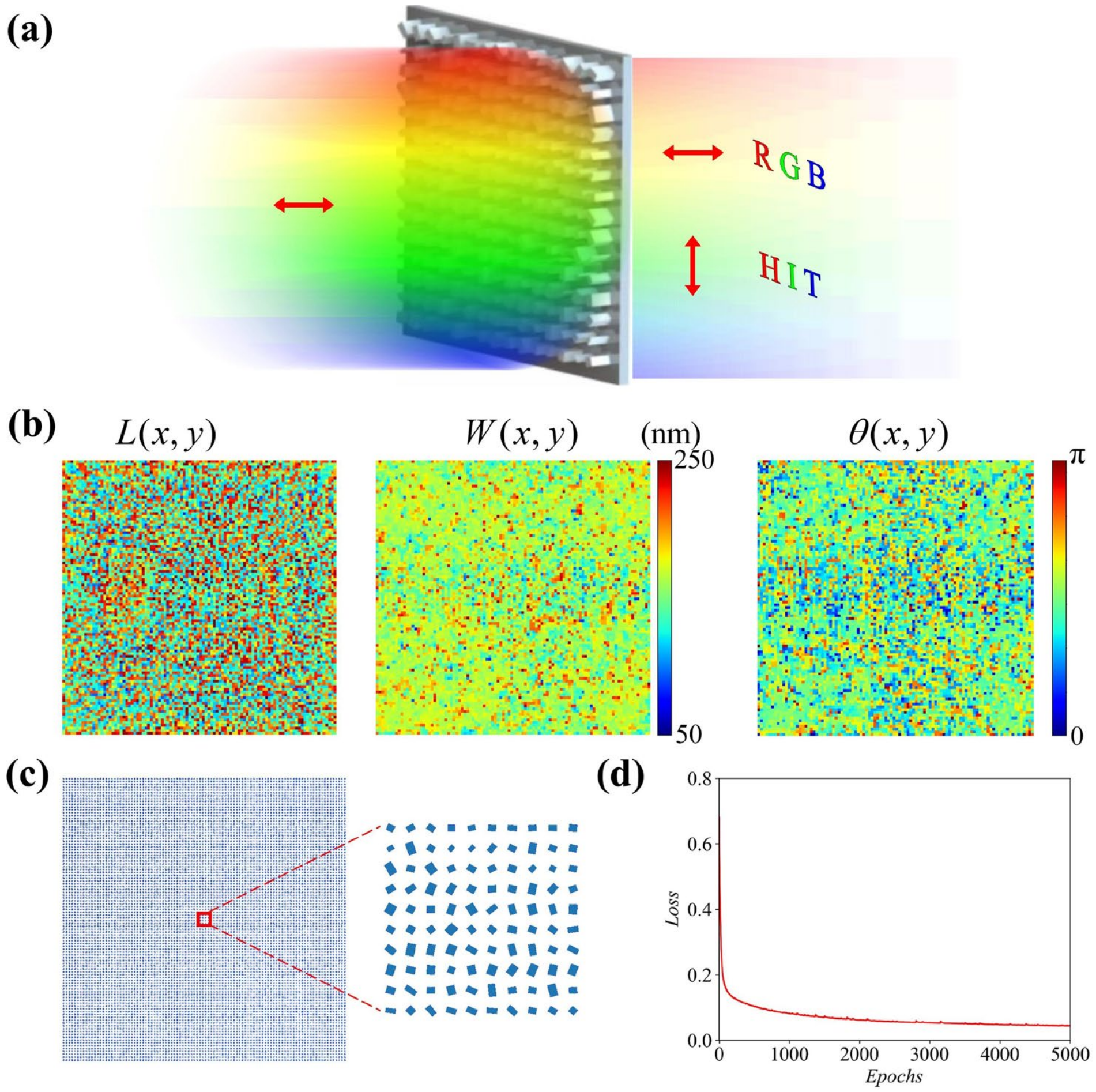

Figure S5. FDTD full-wave simulation of a two-polarization color holographic display. (a) Schematic diagram of wavelength and polarization multiplexing. (b) Distribution map of length (L), width (W), and angle of orientation (θ) of the inversely designed metasurface. (c) Optimized geometric layout of the metasurface. (d) Loss curve during the training process.

Subsequently, a FDTD full-wave simulation was performed based on the meta-atom layout of the optimized metasurface. Plane waves are used to illuminate the metasurface. Field monitors were placed $10\ \text{nm}$ below the metasurface to capture the scattered fields. Then, the near-field data extracted by FDTD was used to compute the holographic images in the target plane via near-to-far-field transformation algorithms. Figure S6(a) displays the target holograms, the outputs from the physical model, and those from FDTD full-wave simulations, respectively. Compared with the physical model outputs, the holographic images from FDTD exhibit a high signal-to-noise ratio. Figure S6(b) shows

the discrete-channel display images of the physical model outputs and the FDTD simulation holography. The PSNRs of these images relative to the target image are labeled in the figure. FDTD simulation results demonstrate the accuracy and reliability of our proposed inverse design method, meta-diff-DONN, for wavelength and polarization multiplexed holography.

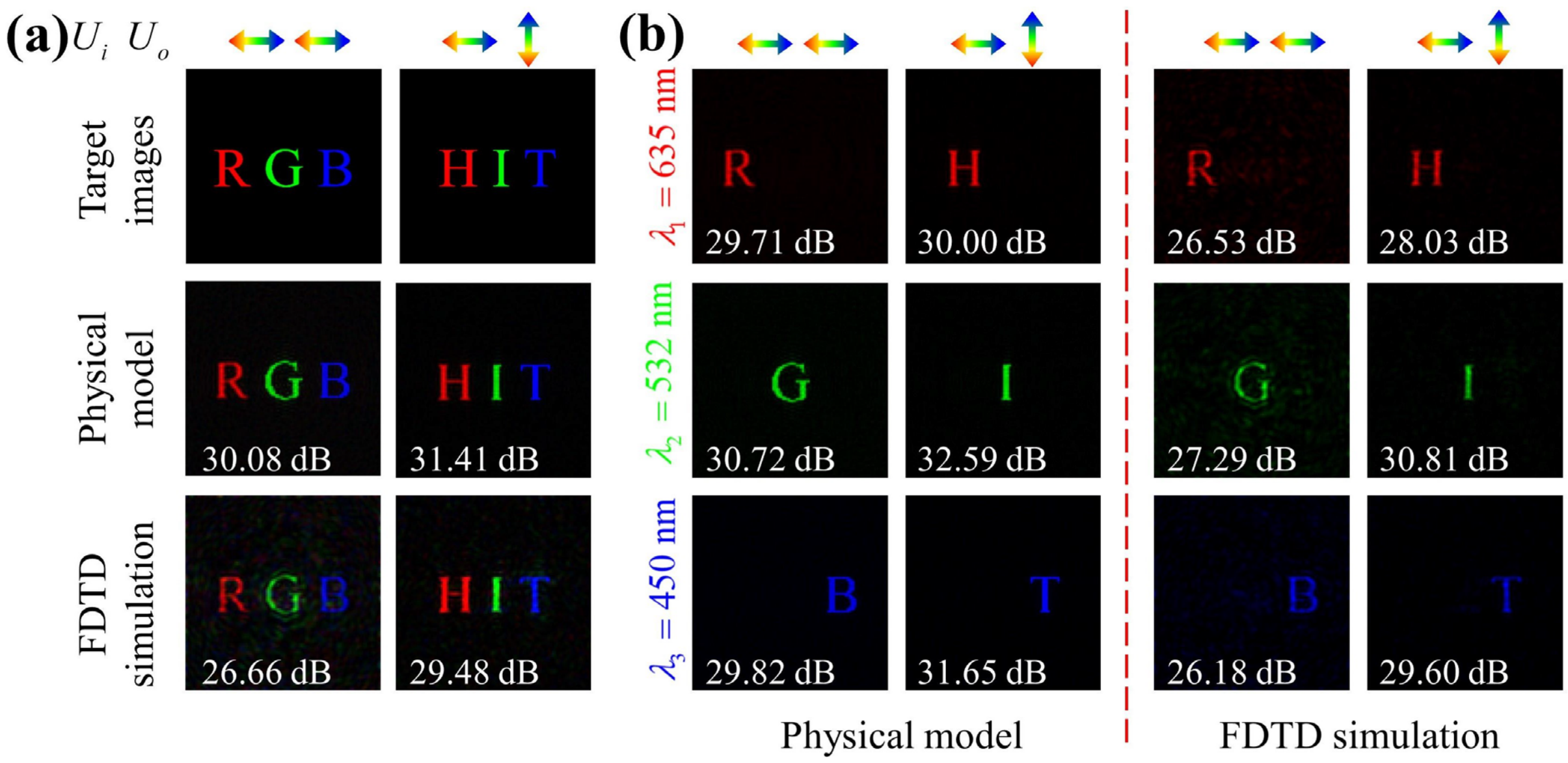

Figure S6. (a) Target, physical model output, and FDTD full-wave simulation color holographic images of the letters "RGB" and "HIT" under different output polarizations. (b) Individual holographic images output from the six holographic channels of the physical model output and full-wave FDTD simulation. Their PSNRs with respect to the target image are annotated in the figure.

Supporting Note S9. Implementation details of the metasurface for polarization and rotation angle multiplexing

A. Meta-diff-DONN for high-resolution image end-to-end inverse design

This case is used for simulation verification of the end-to-end inverse design of high-capacity holographic storage metasurfaces. The target images are sourced as follows:

"Countdown": https://www.aigei.com/item/countdown_timer_1.html (CC0 1.0 Public Domain Dedication, https://creativecommons.org/publicdomain/zero/1.0/),

"A": https://www.aigei.com/item/zi_mu_a_yu_yan.html (CC0 1.0 Public Domain Dedication),

"B": https://www.aigei.com/item/zi_mu_b_yu_yan.html (CC0 1.0 Public Domain Dedication),

"C": https://www.aigei.com/item/zi_mu_c_yu_yan.html (CC0 1.0 Public Domain Dedication).

We employ a two-layer metasurface to achieve polarization and rotation angle multiplexed holography. Each layer consists of a circular metasurface composed of 500×500 ($200\ \mu\text{m}\times200\ \mu\text{m}$)

meta-atoms. The spacing between the two metasurface layers is $d = 230\ \mu m$, and the diffraction distance from the second metasurface to the output plane is $z = 230\ \mu m$. Despite the microscale separation requirement between metasurfaces, two experimental implementation approaches can be developed: (1) Optimal direct configuration: both metasurfaces are mounted substrate-outward [Figure S7(a)]. The 230 μm face-to-face separation can be precisely achieved through micrometer-scale adjustments via the precision displacement stage. (2) Relay-enhanced configuration: it is possible to employ a 4f optical relay system [see Figure S7(b)] to extend the effective working distance along the optical axis. Crucially, the 230 μm distance between the image plane of the primary metasurface and the front facet of the secondary metasurface can be maintained. Although this separation remains microscopic, it was accurately implemented and stabilized using a dual-CCD real-time calibration module. This dual-CCD approach enables practical rotational adjustment of the secondary metasurface.

To physically realize the rotational cascaded metasurfaces with a 230 μm separation, two experimental configurations are proposed in Figure S6. While these setups are theoretically equivalent under ideal paraxial conditions, they present distinct advantages and limitations in practical measurement scenarios.

Configuration (a), the optimal direct face-to-face setup, provides the truest representation of the theoretical physical model. It relies on pure free-space propagation, thereby eliminating insertion losses from additional optical elements and avoiding lens-induced aberrations (such as spherical or chromatic aberrations). However, mechanically implementing this configuration is extremely challenging. Integrating a precision rotary stage for the secondary metasurface within a microscopic 230 μm gap poses a severe risk of mechanical collision and alignment instability during dynamic rotation.

Configuration (b), the relay-enhanced setup, effectively resolves these mechanical constraints by employing a 4f optical relay system. This configuration projects the output plane of the first metasurface to a macroscopic distance, providing ample physical space to securely mount a motorized rotation stage and a dual-CCD real-time calibration module. This ensures high structural stability and precision during continuous rotation. The primary disadvantage is that the 4f system acts as a spatial low-pass filter, which is limited by the numerical aperture (NA) of the relay lenses. High-angle diffracted light may be clipped by the limited lens aperture, potentially causing a slight loss of

resolution or edge information in the final holograms, alongside minor insertion losses introduced by the lenses. In our study, Configuration (b) was primarily considered to guarantee mechanical stability and operational feasibility during the continuous rotational multiplexing measurements.

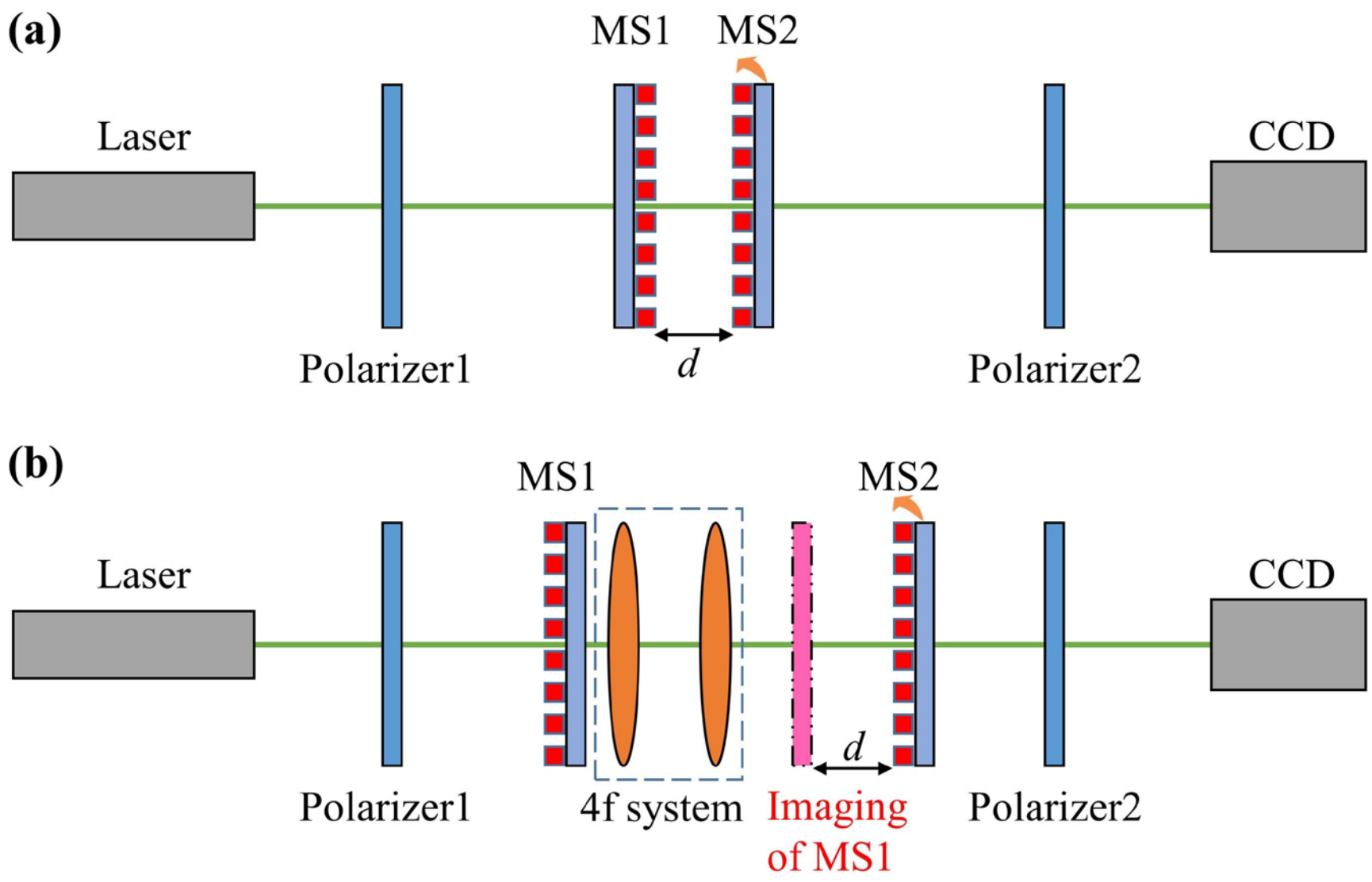

Figure S7. Schematic experimental setups for rotational cascaded metasurface holography. (a) Dual metasurfaces mounted in a directly facing, substrate-outward configuration. (b) Measurement based on an optical 4f relay system (dashed block).

The working wavelength of the incident light is $\lambda = 635$ nm. The first metasurface is fixed, while the second metasurface is rotated counterclockwise at 15° intervals over 24 angles $\delta = 0°, 15°, 30°, ..., 345°$, completing a full rotation. The incident light is *x*-polarized. When the output light is switched to *x*-polarization, and the metasurface rotates uniformly for one full cycle, 24 different monochromatic images are projected, equivalent to a 24 fps video playback for one second. At this time, the "Countdown" video is played. Figure S8 shows a single frame of the holographic video. When the output light is switched to *y*-polarization, and the metasurface rotates uniformly for one full cycle, the "ABC" video is played. Figure S9 displays a single frame of the holographic video. Supporting Video 1 fully demonstrates the output effect of the dual videos. Using the Adam optimizer with a learning rate of 0.01 and training for 2000 epochs, the optimized geometric layouts of the two-layer metasurfaces are shown in Figure S10(a), and the loss curve is illustrated in Figure S10(b).

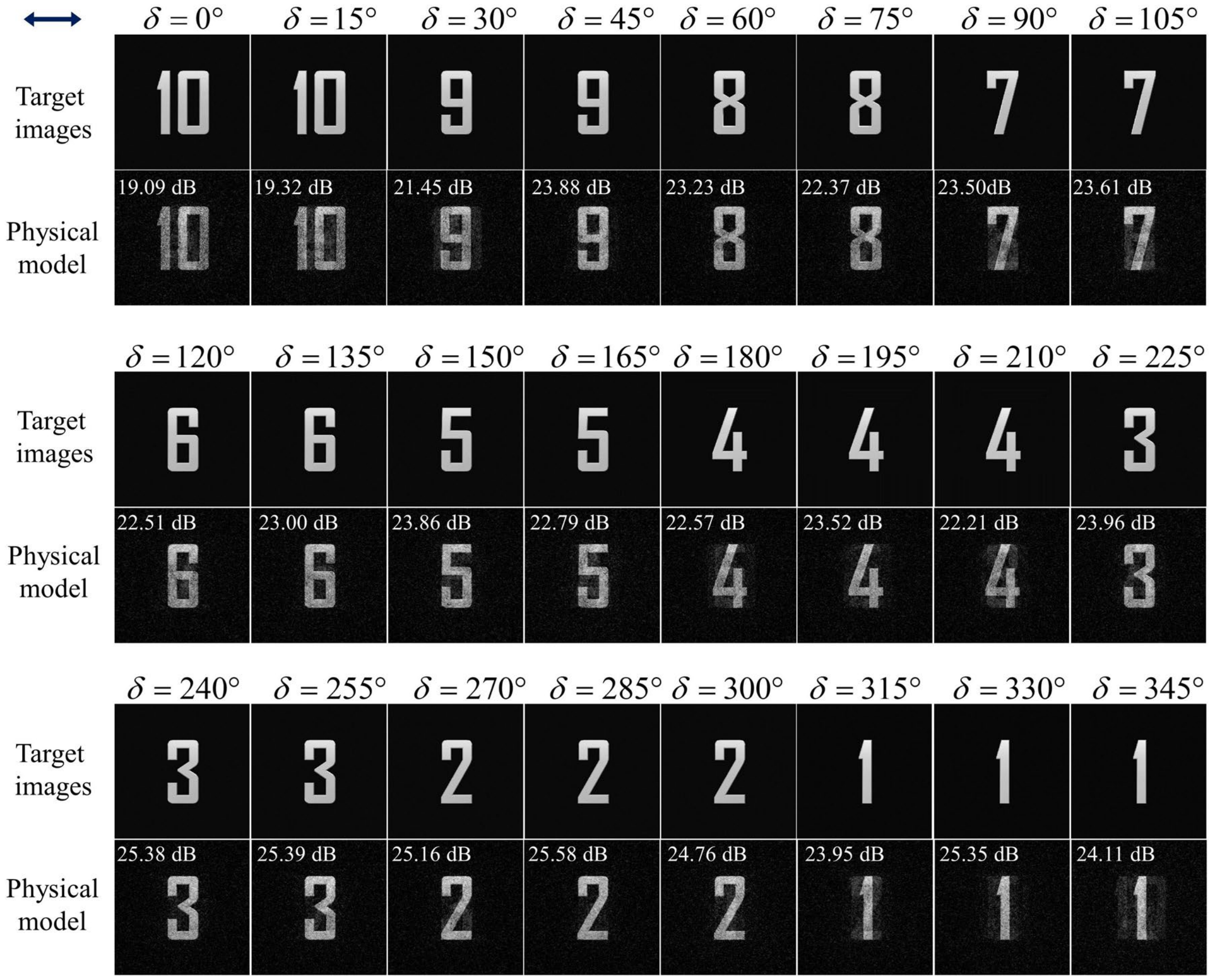

Figure S8. The output light is x-polarized, the second layer of the metasurface rotates uniformly at 15° intervals, and 24 frames of holographic images are detected during one full rotation.

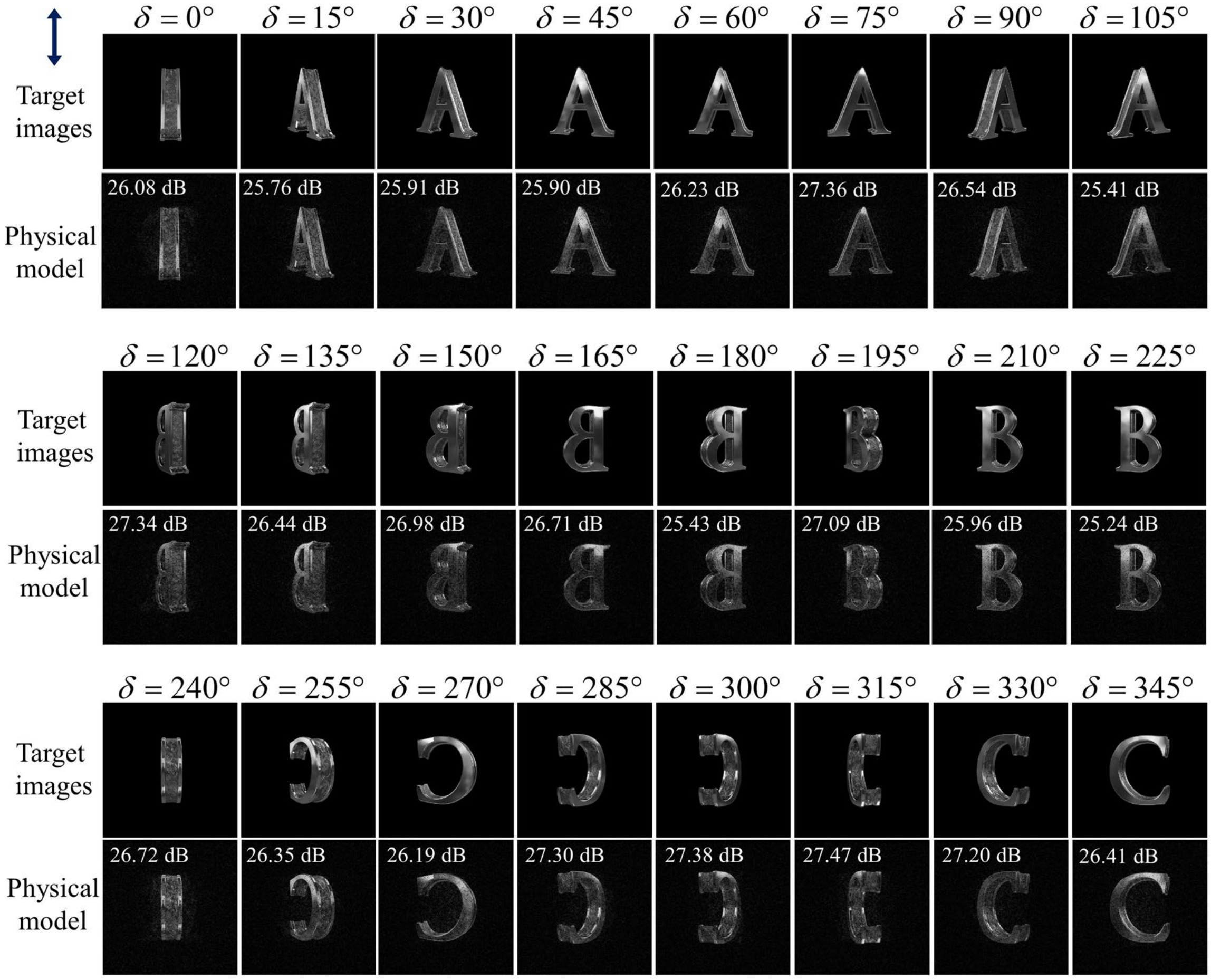

Figure S9. The output light is *y*-polarized, the second layer of the metasurface rotates uniformly at 15° intervals, and 24 frames of holographic images are detected during one full rotation.

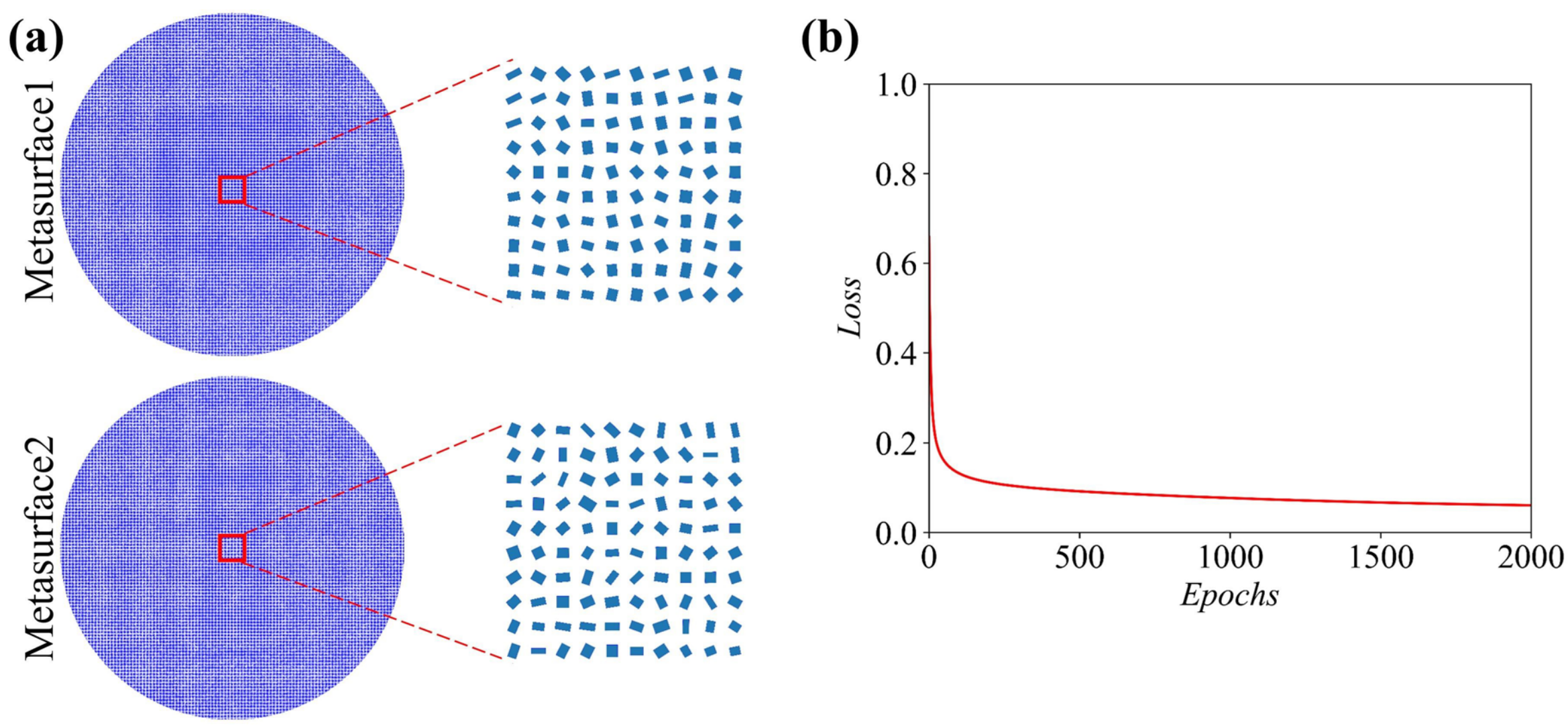

Figure S10. Polarization and rotation angle multiplexing holography. (a) Optimized two-layer metasurface structure. (b) Loss curve during optimization of polarization and rotation angle multiplexing holography.

B. The upper limit of frame rate in holographic video

The interdependence between holographic channels realized at different twist angles of the second-layer metasurface imposes a fundamental constraint on the achievable frame rate in holographic video applications. Experimentally, however, metasurface rotation is readily achievable using a single high-precision coaxial mechanism (e.g., the LETEK RP-1A rotary stage, capable of 360° continuous rotation with an angular sensitivity of 1°).

To evaluate the possible upper limit of angular multiplexing for the second metasurface under single-wavelength, single-polarization illumination, we have maintained the fixed metasurface dimensions and unit cell counts (consistent with the main text: a two-layer metasurface, each layer comprising a 500 × 500 meta-atom array, with both the inter-layer separation and the distance from the second metasurface to the detector set to 230 μm) and conducted inverse optimization for 360 channels (1° step), 180 channels (2° step), and 90 channels (3° step). These configurations yielded holograms with relatively low image quality (average PSNR~10 dB). Acceptable holographic image quality was achieved with 60 channels (6° step), resulting in an average PSNR of 24.21 dB (see Figure S11). Our analysis indicates that a 6° step (60 angles per 360°) would likely represent the achievable angular resolution limit under the current design parameters. The limitation on further angular resolution enhancement would depend on several factors: the total number of multiplexed channels, the finite metasurface modulation capability, the image quality requirement, and the computational resources.

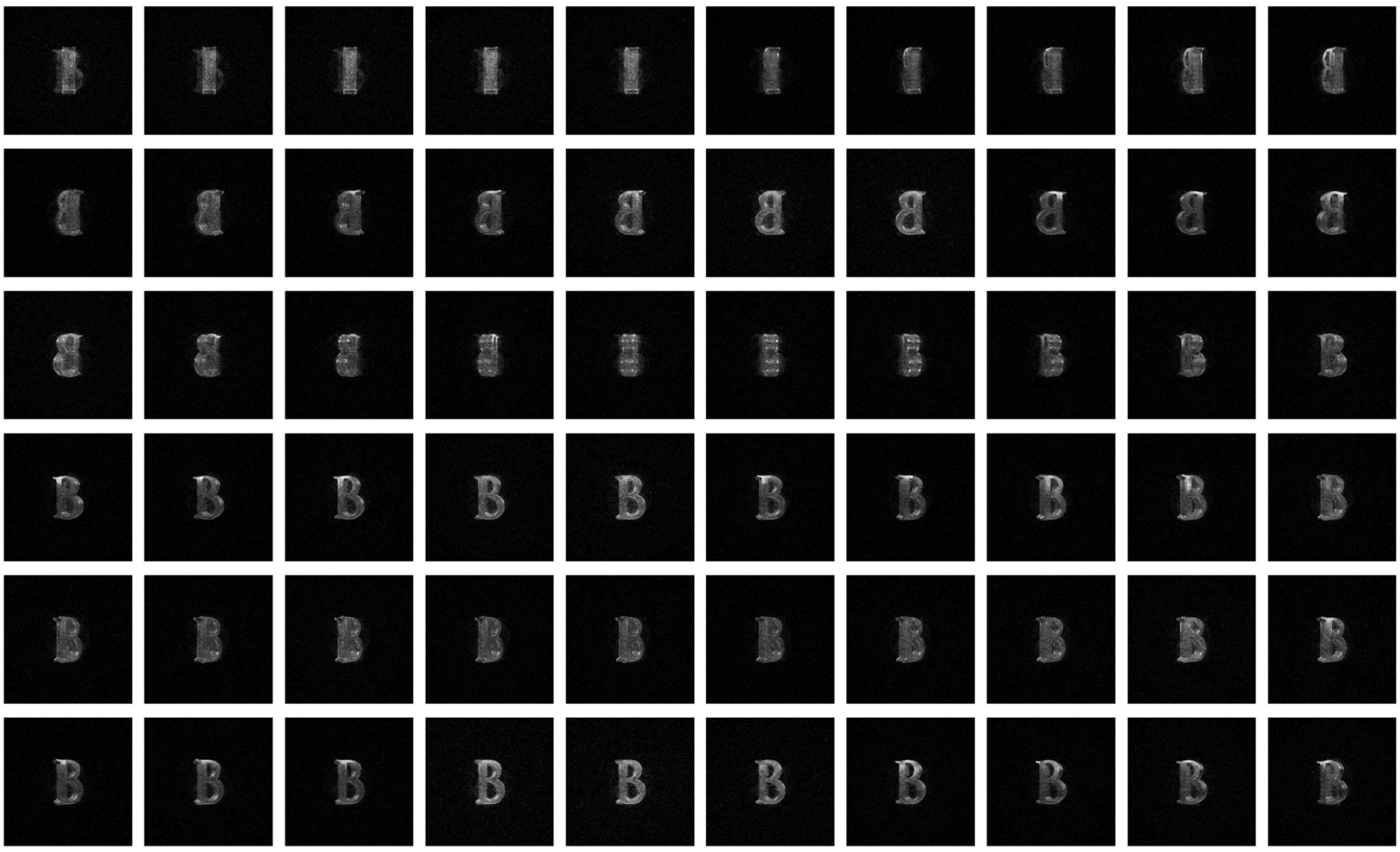

Figure S11. Holographic images generated by the second metasurface under single-wavelength *x*-polarized illumination at 60 rotational positions with 6° intervals.

Supporting Note S10. Implementation details of metasurface for wavelength, polarization, and rotation angle multiplexing co-design

A. Meta-diff-DONN for high-resolution image end-to-end inverse design

This case is used to simulate and verify the performance of the meta-diff-DONN for end-to-end inverse design of high-capacity holographic storage metasurfaces. The target images used for this simulation are sourced as follows:

"Flame1" by Dao le ge xi gua: https://www.aigei.com/item/mg_dong_hua_huo.html (CC BY 4.0 https://creativecommons.org/licenses/by/4.0/)

"Flame2": https://www.aigei.com/item/fa_guang_huo_ya_2.html (CC0 1.0 Public Domain Dedication, https://creativecommons.org/publicdomain/zero/1.0/)

"Yellow flower": https://www.aigei.com/item/flowers_product.html (CC0 1.0 Public Domain Dedication)

"Colorful butterfly": https://www.aigei.com/item/san_zhi_ke_ai_d_18.html (CC0 1.0 Public Domain Dedication)

"Blue butterfly": https://www.aigei.com/item/blue_butterflie.html (CC0 1.0 Public Domain Dedication)

"Fish": https://www.aigei.com/item/dong_man_ka_ton_2196.html (CC0 1.0 Public Domain Dedication)

The overall architecture includes two layers of circular metasurfaces, each composed of 500×500 meta-atoms occupying an area of $200 \times 200\ \mu\text{m}^2$, with two angular spectrum propagation diffraction distances both set to $z = 230\ \mu\text{m}$. The size of the meta-atoms is also set to $P = 400$ nm, and the RGB wavelengths used are $\lambda_1 = 450$ nm, $\lambda_2 = 532$ nm, and $\lambda_3 = 635$ nm. The second layer of the metasurface rotates counterclockwise through 24 angles at 15° intervals, completing a full rotation. Supporting Video 2 illustrates four different holographic videos played under four polarization conversion scenarios. Figures S12-S15 depict the complete sets of 24 frames of full-color holographic images stored in the *x-x, x-y, y-x*, and *y-y* polarization conversion channels, respectively. The optimized length (L), width (W), orientation angle (θ) distributions, and geometric layouts of the two layers of metasurfaces using the meta-diff-DONN are shown in Figures S16(a) and S16(b). Figure S16(c) presents the loss curve during the end-to-end inverse optimization iteration process.

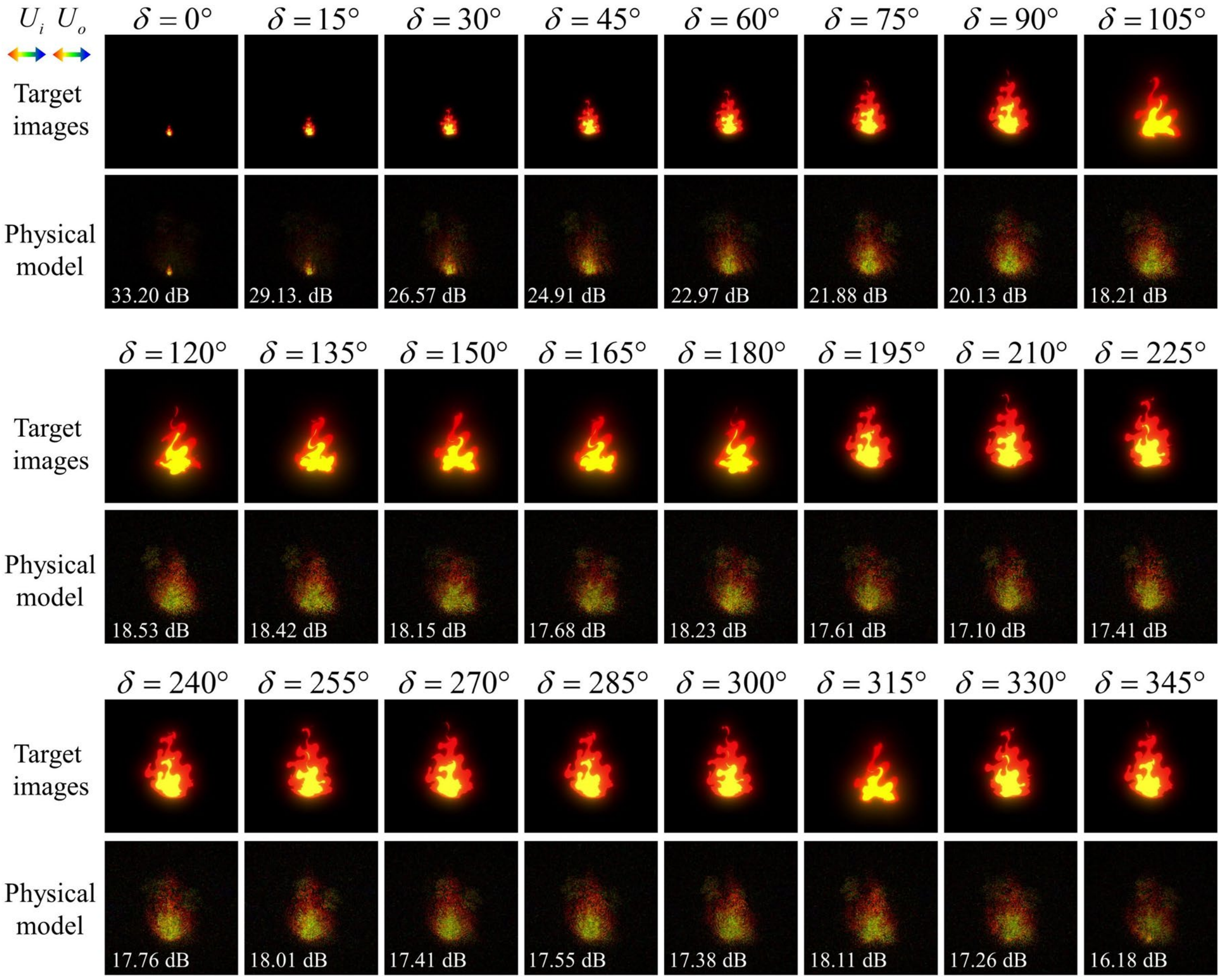

Figure S12. For the "*x*-*x*" polarization conversion channel, with the first layer metasurface fixed, the second layer metasurface is rotated counterclockwise in 15° intervals, capturing 24 frames of color images on the output plane.

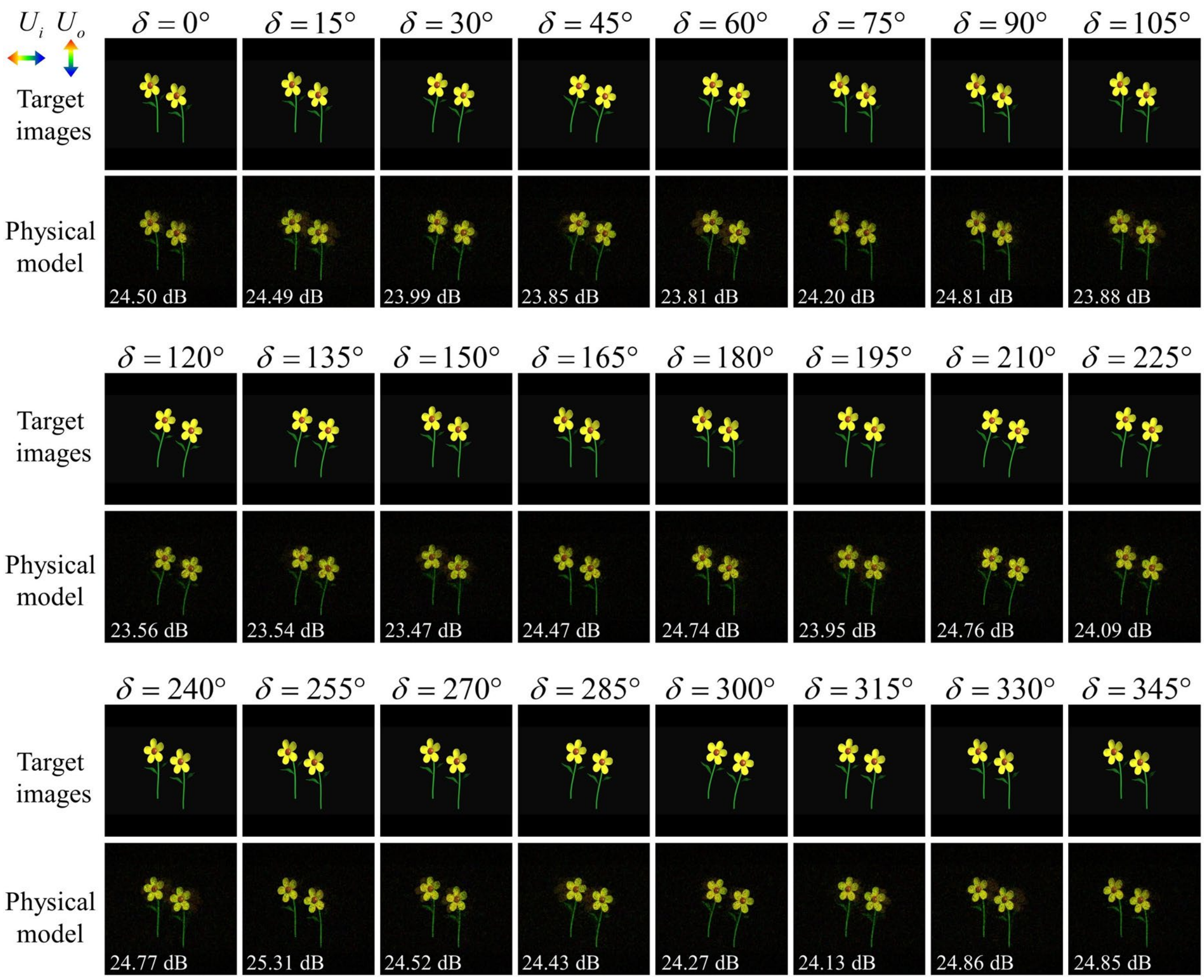

Figure S13. For the "*x*-*y*" polarization conversion channel, with the first layer metasurface fixed, the second layer metasurface is rotated counterclockwise in 15° intervals, capturing 24 frames of color images on the output plane.

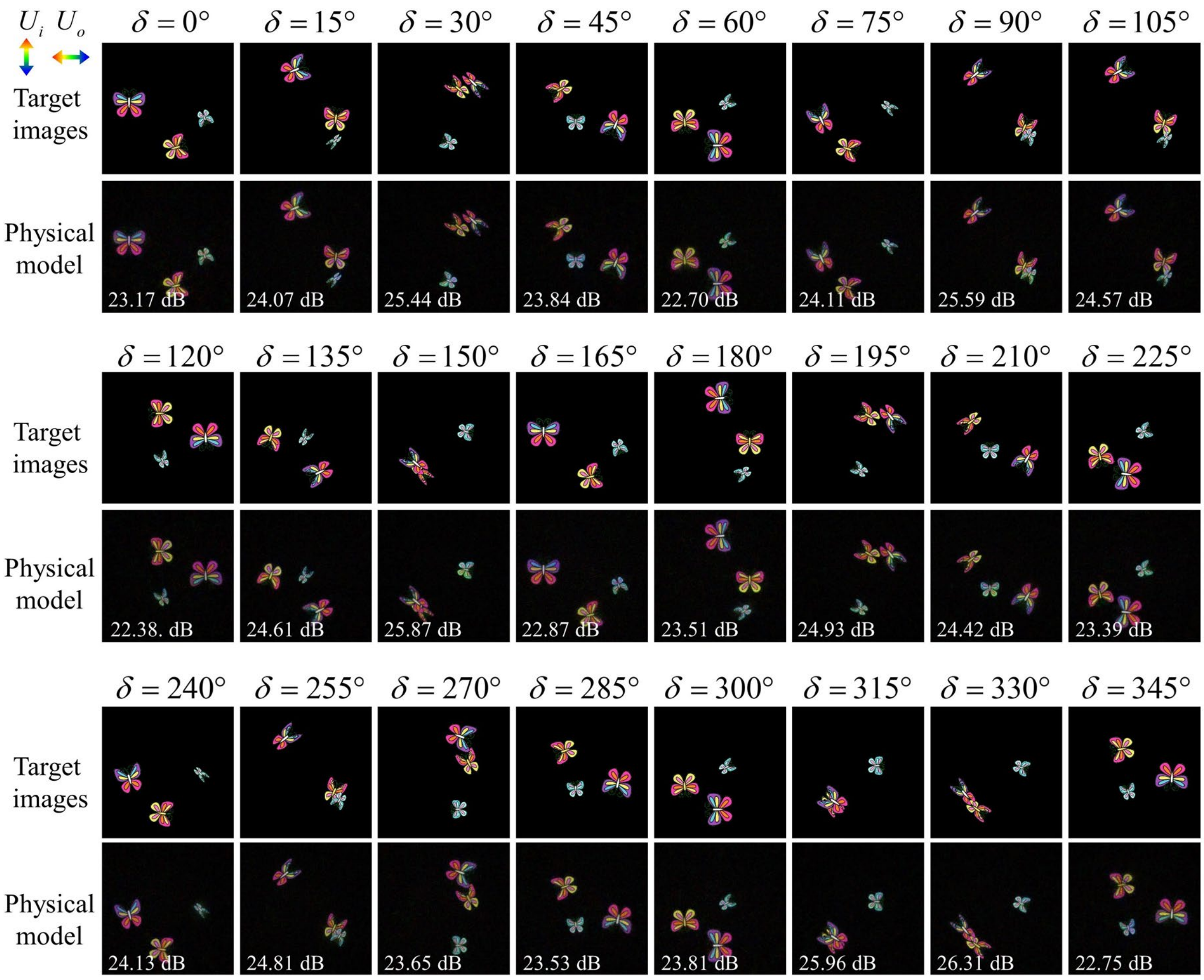

Figure S14. For the "*y*-*x*" polarization conversion channel, with the first layer metasurface fixed, the second layer metasurface is rotated counterclockwise in 15° intervals, capturing 24 frames of color images on the output plane.

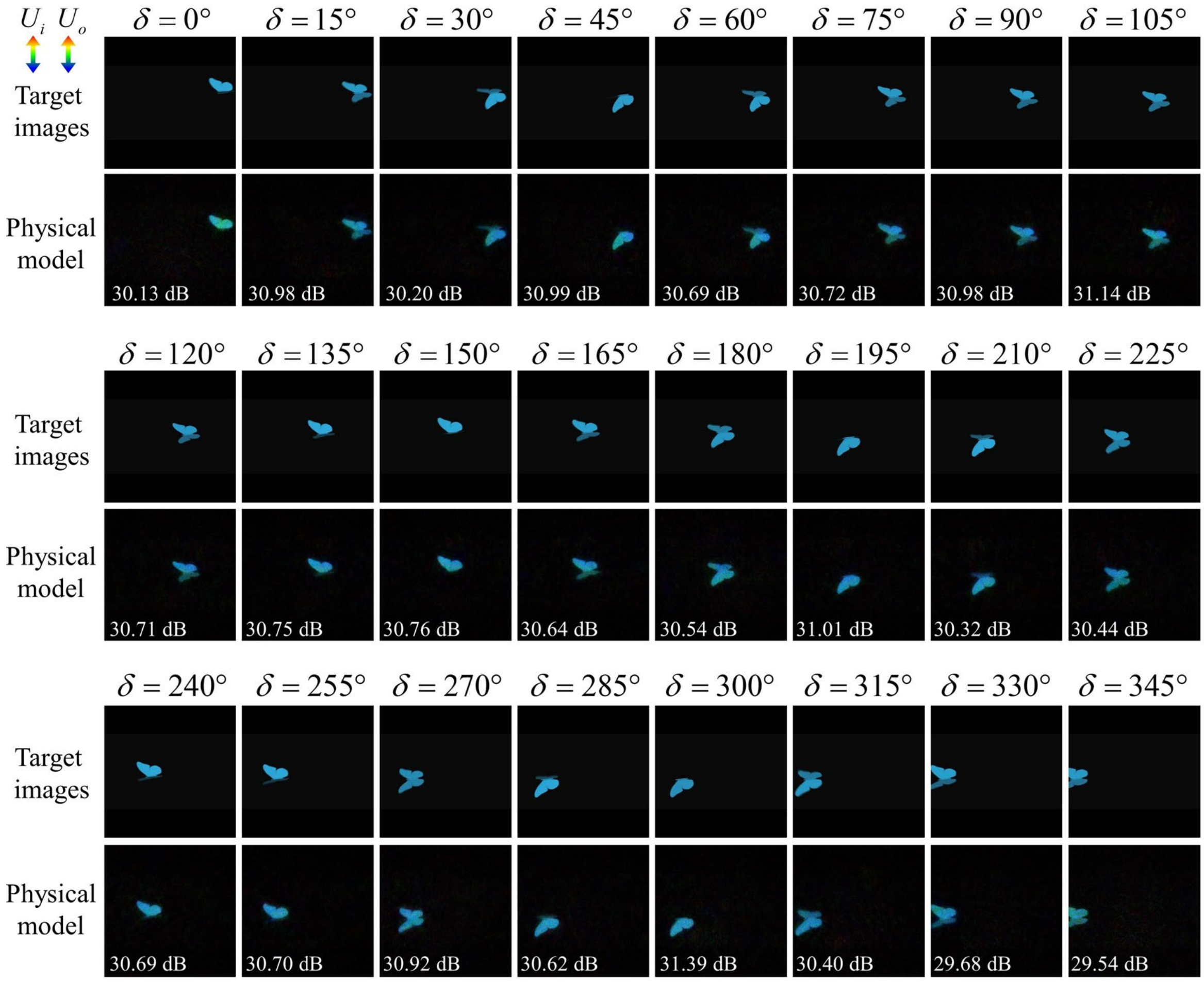

Figure S15. For the "*y*-*y*" polarization conversion channel, with the first layer metasurface fixed, the second layer metasurface is rotated counterclockwise in 15° intervals, capturing 24 frames of color images on the output plane.

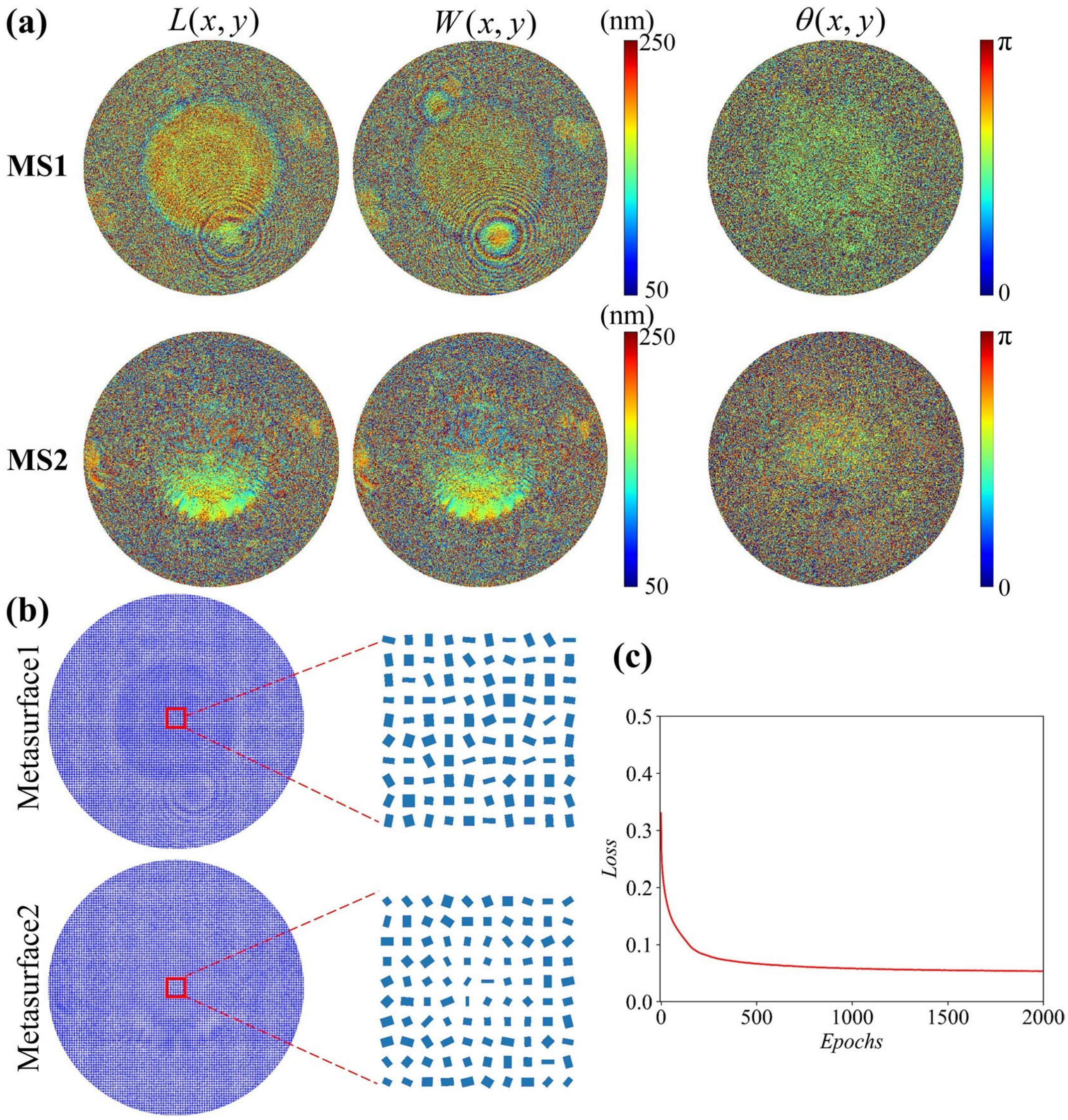

Figure S16. Wavelength, polarization, and rotation angle multiplexing holography. (a) The length (L), width (W), and orientation angle (θ) distribution diagrams of the optimized double-layer metasurface. (b) The optimized double-layer metasurface structure. (c) The loss curve of the training process.

B. FDTD validation of the proposed methodology on a small-scale model

We also employed FDTD full-wave simulations to validate the effectiveness of our proposed twist-metasurface end-to-end design framework. We used a two-layer 100×100 -unit ($40\times40\ \mu\text{m}^2$) metasurface with a spacing between the two metasurfaces and a diffraction distance from the metasurface to the output plane set to $d = z = 60\ \mu\text{m}$ (see Figure S17(a)). The input light was *x*-polarized, and by switching the output polarization states, we obtained color holographic images of

letters “RG” and “ab”. We further rotated the second metasurface by two angles $\delta = 60°, 120°$ and obtained color holographic images of letters “BHIT” and “cdef”. Figure S18(a) shows the optimized length, width, and orientation angle distributions of the metasurface based on the end-to-end design method, while Figure S18(b) illustrates its geometric layout. The loss curve during training is plotted in Figure S18(c).

The field distribution at the front interface of the second-layer metasurface, obtained from direct full-wave simulations, was numerically archived and subsequently utilized as the excitation source for downstream layers.

Subsequently, we performed FDTD full-wave simulations based on the optimized meta-atom layout. Given the computationally intensive and memory-demanding nature of full-wave FDTD simulations for bilayer metasurfaces, we adopted a modular simulation strategy constrained by hardware limitations. First, we simulate the far-field of the two-color plane wave passing through the first metasurface, in which a field monitor was placed at a distance z=60 μm below the metasurface to capture the scattered field. The optical field distribution at the front surface of the second layer was implemented as a customized source to calculate the scattered field through the secondary metasurface, obtained nearfield of secondary metasurface. Then, the holographic image at the output plane can be calculated using a near-field to far-field transformation algorithm. Subsequent investigations involved rotating the secondary metasurface by 60° or 120° while maintaining identical computational methodology, with the resultant holographic patterns presented in Figure S17(b). The PSNRs of the physical model outputs and the FDTD simulation results, each relative to the target image, are annotated in the figure. Notably, the FDTD-simulated holograms showed lower quality than the physical model outputs but remained recognizable target images. The consistency across the two computed results demonstrates the accuracy and reliability of our proposed end-to-end inverse design method, meta-diff-DONN, for wavelength, polarization, and rotation angle multiplexed holography.

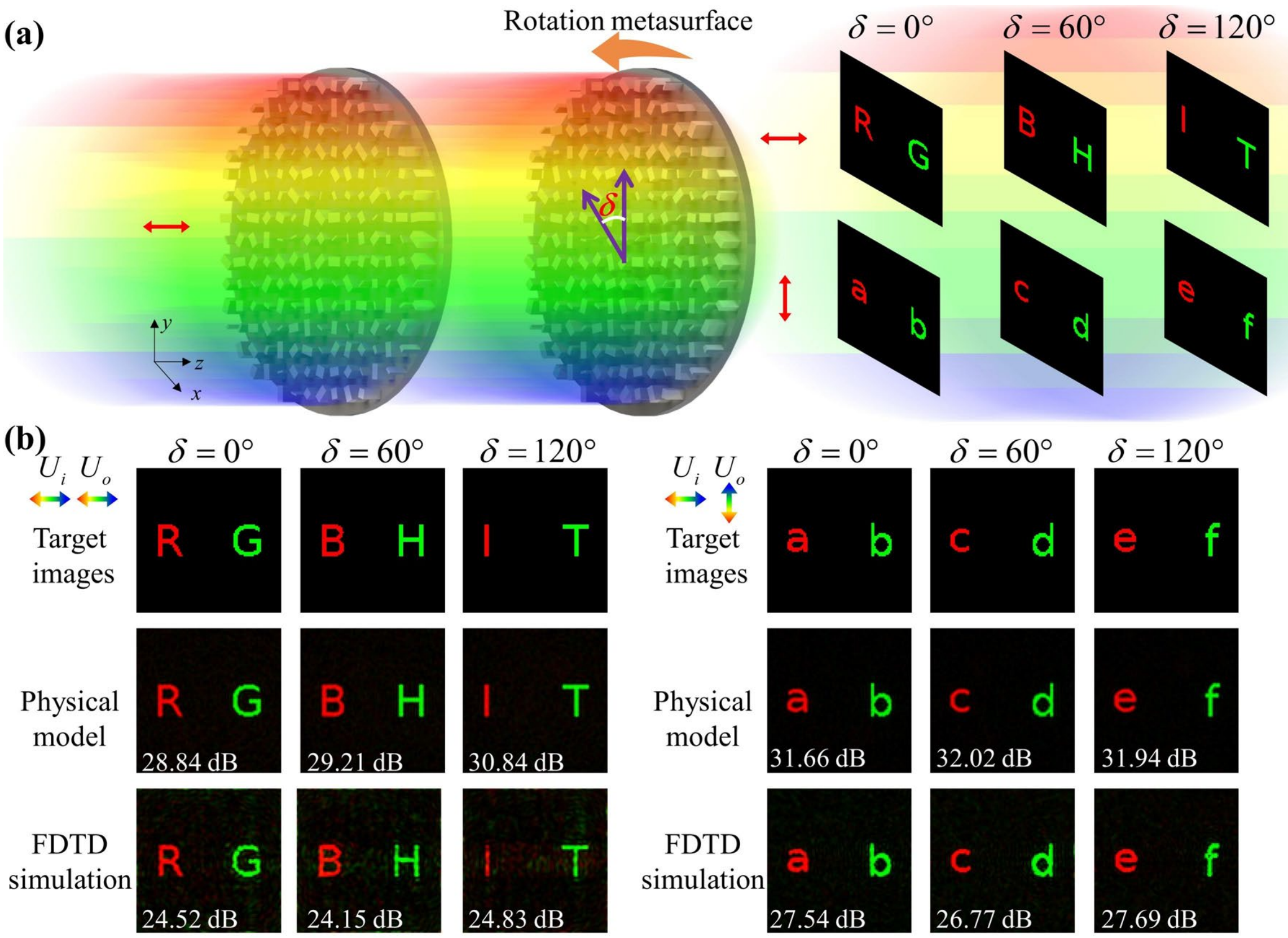

Figure S17. The FDTD full-wave simulation for wavelength, polarization, and rotation angle multiplexed holography. (a) Schematic of wavelength, polarization, and rotation angle multiplexing. (b) The target images, physical model outputs, and FDTD full-wave simulated color holographic images under different output polarization states and rotation angles. In this figure, red images represent holographic images under 635 nm illumination, green images represent those under 532 nm illumination, and the PSNRs of these images relative to the target images are labeled accordingly.

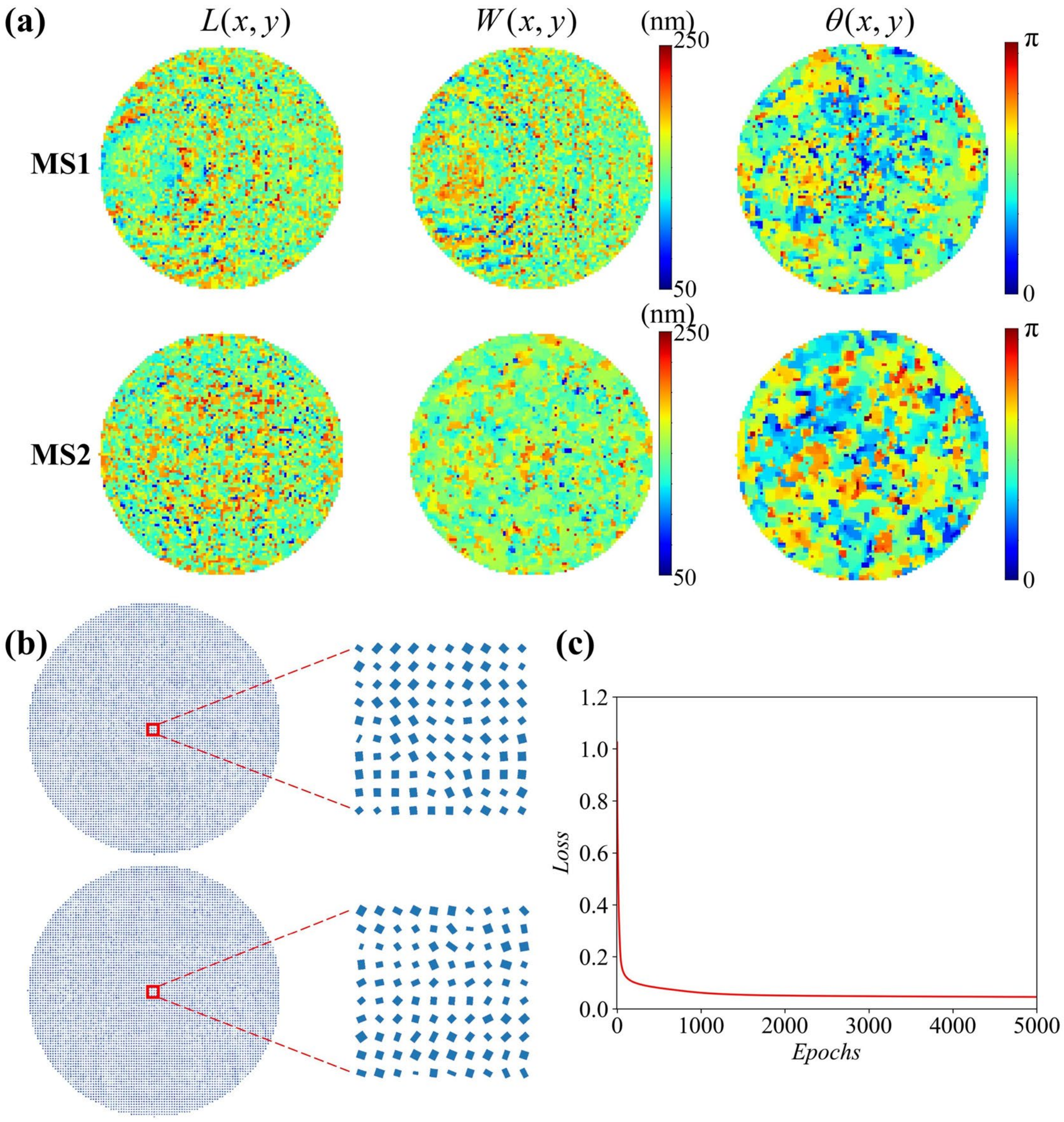

Figure S18. Small-scale metasurface optimized process for wavelength, polarization, and rotation angle multiplexed holography. (a) Inverse design results about the length, width, and angle of the orientation distribution map of the metasurfaces. (b) The optimized geometric layout of the metasurfaces. (c) The loss curve during the training process.

Supporting Note S11. Assessment of the impact of experimental error on the metasurface performance

A degree of mismatch exists between the simulated design and its physical realization, primarily stemming from two sources: (1) fabrication imperfections: Deviations in the geometric dimensions (e.g., length, width) and orientation angles of the constituent meta-atoms during the manufacturing process. (2) Experimental alignment challenges: Difficulties in precise alignment during assembly and

experimentation, including lateral displacement, tilt (rotation about the *x*- or *y*-axis), and rotational misalignment between metasurface layers. These imperfections collectively degrade the quality of the resulting holographic images.

To test the robustness of our designed metasurface to fabrication errors, we introduced manufacturing shape error noise into the geometric layout of the metasurface. Based on the optimized metasurface in the main text Section 3.1, we respectively applied ±5% and ±10% Gaussian-distributed random machining error perturbations, which were normalized to fit the dimensional range of meta-atoms. The length (L) and width (W) of meta-atoms range from 50 nm to 250 nm, meaning the ±5% and ±10% Gaussian white noise levels correspond to fabrication error perturbations of ±10 nm and ±20 nm in meta-atoms' length/width, respectively. Figure S19(a) shows the test results, where the holographic images become increasingly blurred with larger shape errors. Notably, metasurfaces with more layers exhibit more pronounced holographic quality degradation caused by machining shape errors.

During the end-to-end optimization, we introduced 5% Gaussian white noise to simulate geometric errors induced by fabrication limitations, thereby enabling robustness training against noise. In testing, ±5% and ±10% Gaussian noise were separately added to the optimized metasurfaces' length (L) and width (W). As shown in Figure S19(b), the holographic images maintained consistent quality without significant degradation under varying error levels.

When noise was incorporated during training, the PSNR of color holographic images remained stable under shape error variations. In contrast, PSNR became highly sensitive to such variations without noise incorporation. Thus, by integrating shape error noise into the end-to-end design framework, we achieved a metasurface robust to manufacturing imperfections.

To further elucidate the impact of experimental errors on cascaded metasurfaces, we analyzed the effect of meta-atom fabrication defects (length, width, and orientation angle deviations) on the 288-channel hologram performance presented in Section 3.3 (see Figure S21 (a)). Subsequently, the influence of experimental alignment errors was investigated, including lateral offset (Figure S22(a)), rotational misalignment (Figure S23(a)), and tilt about the x- or y-axis (Figure S24(a)). These analyses demonstrate that the end-to-end optimized metasurfaces exhibit notable robustness against fabrication errors encountered in practical experiments.

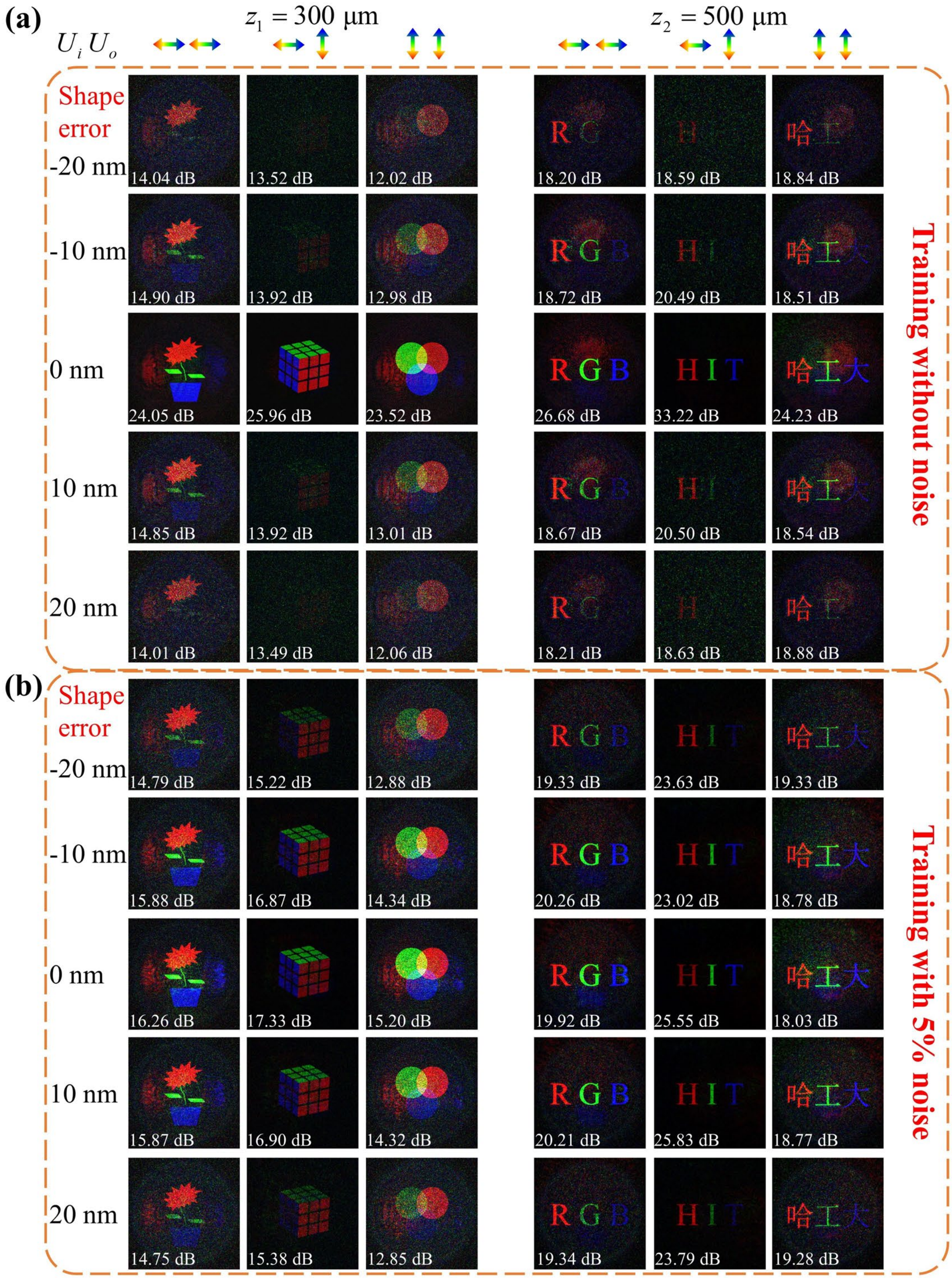

Figure S19. Testing the impact of processing shape errors of meta-atoms on 18-channel holography. (a) No noise was added during the training process, and (b) 5% Gaussian noise was added during the training process.

To bridge the gap between simulations and experiments and enhance the robustness of the designed metasurfaces, we propose several strategies, which are integrated into the meta-differentiable DONN inverse optimization framework. First, we explored the introduction of perturbations to

improve the robustness of holographic metasurfaces. Fabrication imperfections of the metasurface, such as deviations in the length, width, and orientation of meta-atoms, were modeled as Gaussian noise. Similarly, experimental misalignment errors were represented using Legendre polynomials. By incorporating these perturbations into the meta-diff-DONN optimization process, the trained metasurface parameters exhibit intrinsic immunity to fabrication tolerances and alignment inaccuracies. However, this approach is primarily effective for systems with a relatively small number of channels. For our high-capacity metasurface—employing three wavelengths, four polarization conversions, and 24 rotation angles—the complex multiplexing mechanism induces strong coupling among the optical parameters, making it challenging for conventional optimization methods to achieve low-loss convergence. Sharpness-aware minimization (SAM), proposed by Foret et al. in 2021, is an effective algorithm designed to enhance model generalization[1]. Traditional optimization typically focuses solely on minimizing output error and often converges to sharp local minima. These minima are highly sensitive to parameter perturbations and can lead to significant performance degradation. We incorporated the SAM parameter update mechanism into the meta-diff-DONN optimization loop. SAM not only minimizes the error value but also reduces the "sharpness" of the loss landscape. This approach guides the optimization toward flatter minima, ensuring robust diffraction performance under parameter variations. The parameter update mechanism is illustrated in Figure S20(a). The SAM optimizer is based on the principle that, during each parameter update, the model parameters are not updated solely along the gradient descent direction. Instead, the parameters are first perturbed along the gradient direction in the parameter space, the gradient is computed at this perturbed point, and then the parameters are updated accordingly. This procedure helps the model avoid sharp local minima and enhances its generalization performance. We compared the performance of SAM and conventional BP in optimizing a 288-channel holographic metasurface. Both methods converged after 2000 training epochs, and the corresponding loss curves are shown in Figure S20(b). Figures S20(c) and S20(d) depict the loss landscapes obtained using BP and SAM, respectively. To highlight the maximum curvature, the x- and y-axes represent the two principal directions of the Hessian matrix of the loss function with respect to the optical field, while the intensity corresponds to the loss value. The results indicate that the SAM-trained system exhibits a markedly flatter landscape, demonstrating higher stability and robustness with respect to parameter variations[2]. This robustness is further quantified by the maximum eigenvalue $\lambda_{\max}$ of the Hessian matrix: smaller eigenvalues indicate flatter loss surfaces. Compared with conventional BP, $\lambda_{\max}$ decreases dramatically from 216.46 to 35.27, highlighting the effectiveness of SAM in designing robust devices.

Furthermore, we compared the robustness of metasurfaces optimized via BP and SAM against fabrication errors, lateral shifts, rotational misalignments, and tilt errors (Figures S21–S24). Although

SAM training may slightly reduce the holographic image quality, it substantially enhances tolerance to perturbations, maintaining high holographic fidelity even under significant misalignments.

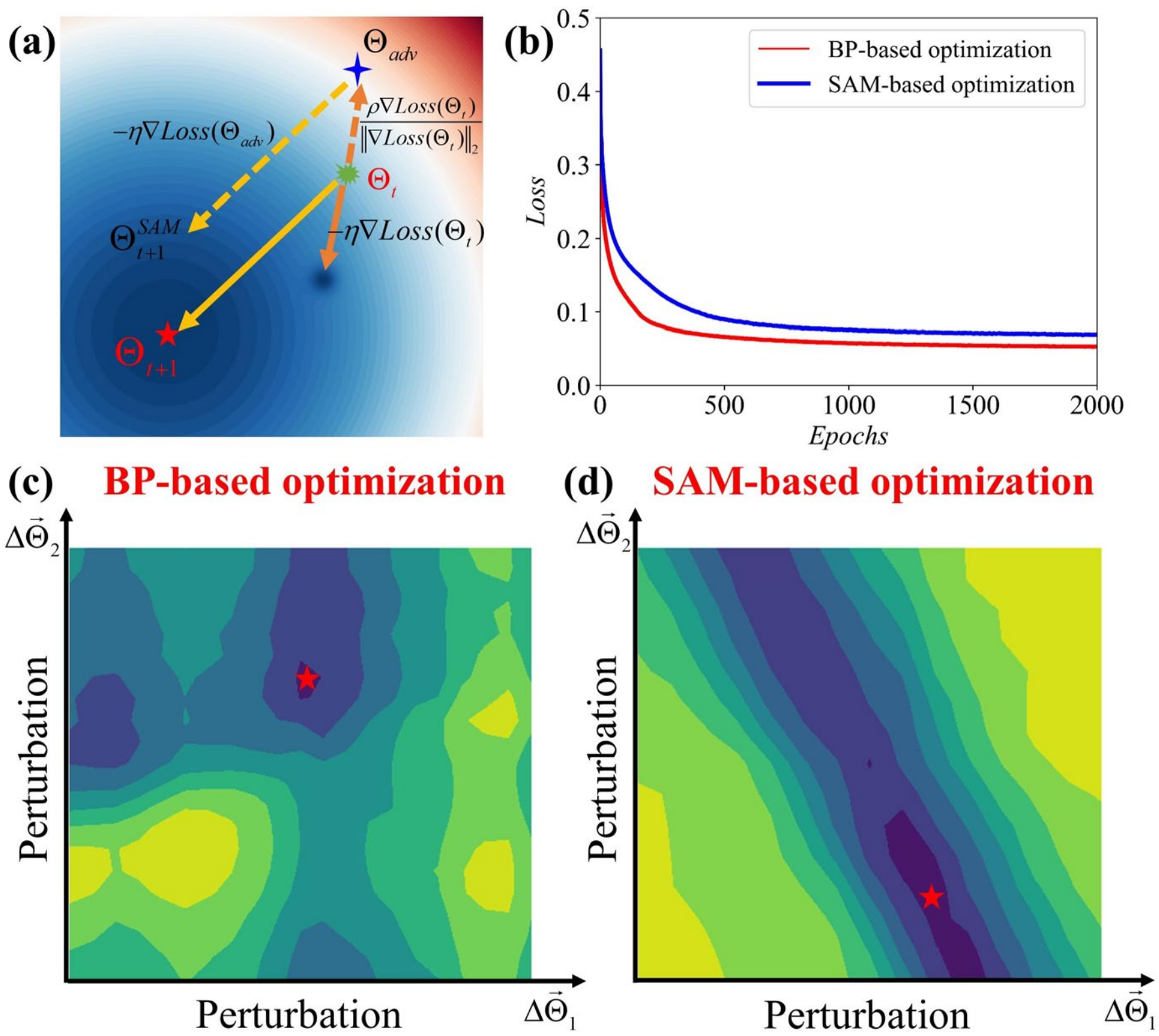

Figure S20. Principle of SAM optimization and comparison with BP optimization. (a) Schematic illustration of the parameter update process in the SAM-based optimization scheme. (b) Convergence curves of the loss function using BP and SAM optimization methods. (c) Loss landscape of the model after BP optimization. (d) Loss landscape of the model after SAM optimization, with asterisks indicating the locations of the minimum loss.

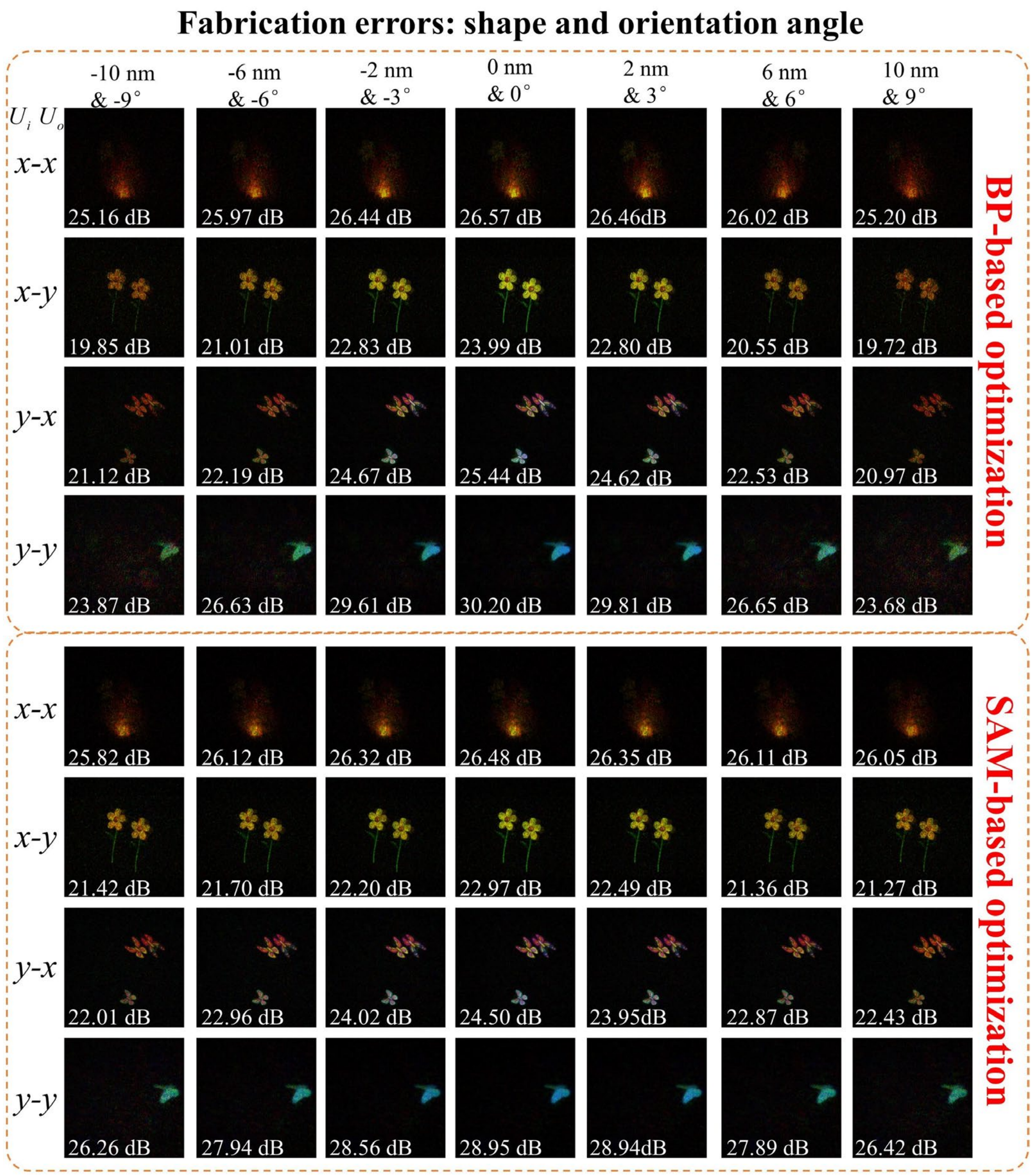

Figure S21. Effects of metasurface fabrication errors in geometry and orientation on 288-channel holography using BP- and SAM-based optimization, respectively.

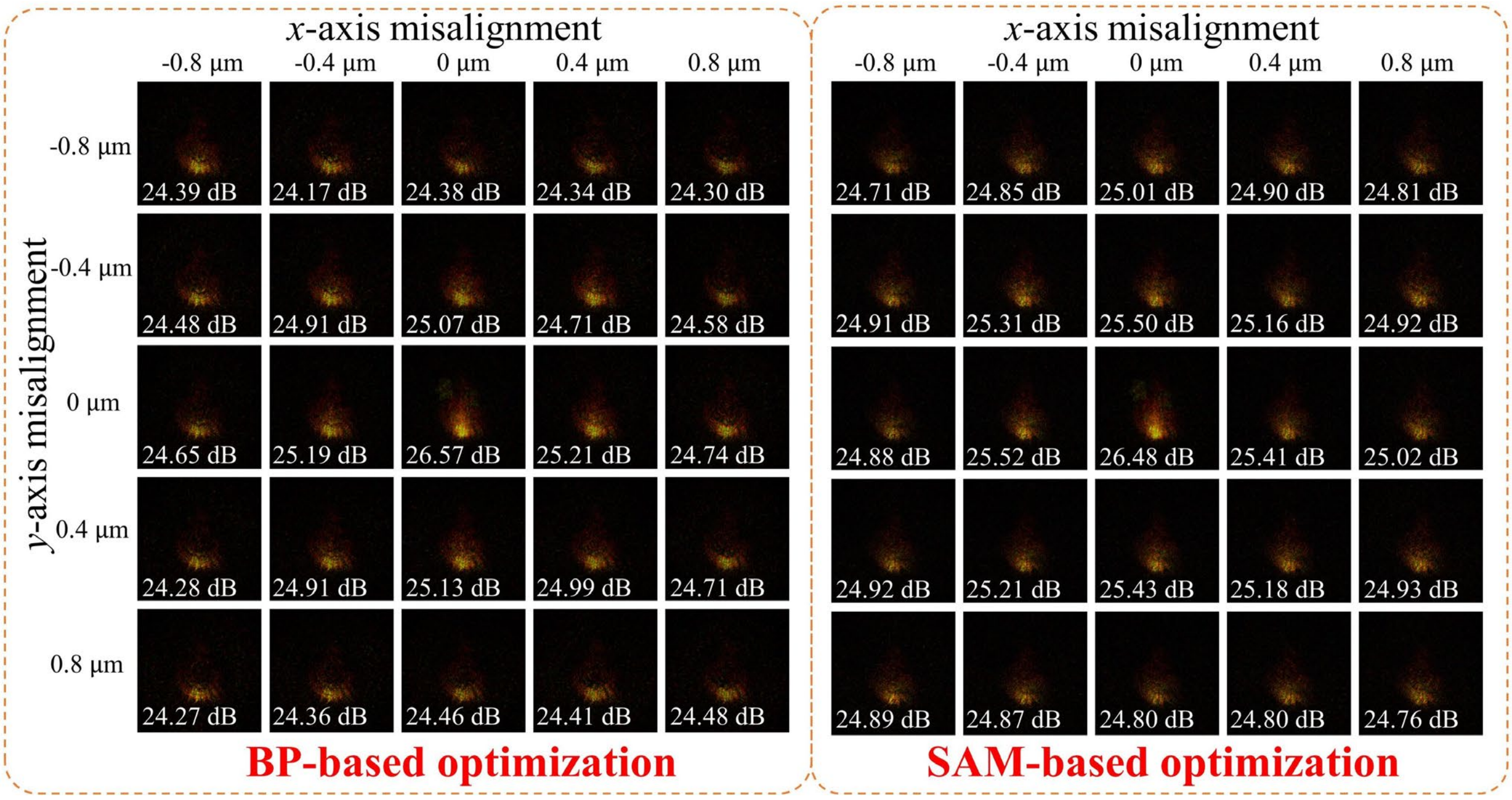

Figure S22. Impact of lateral misalignment in the secondary metasurface on 288-channel holographic performance using BP- and SAM-based optimization, respectively.

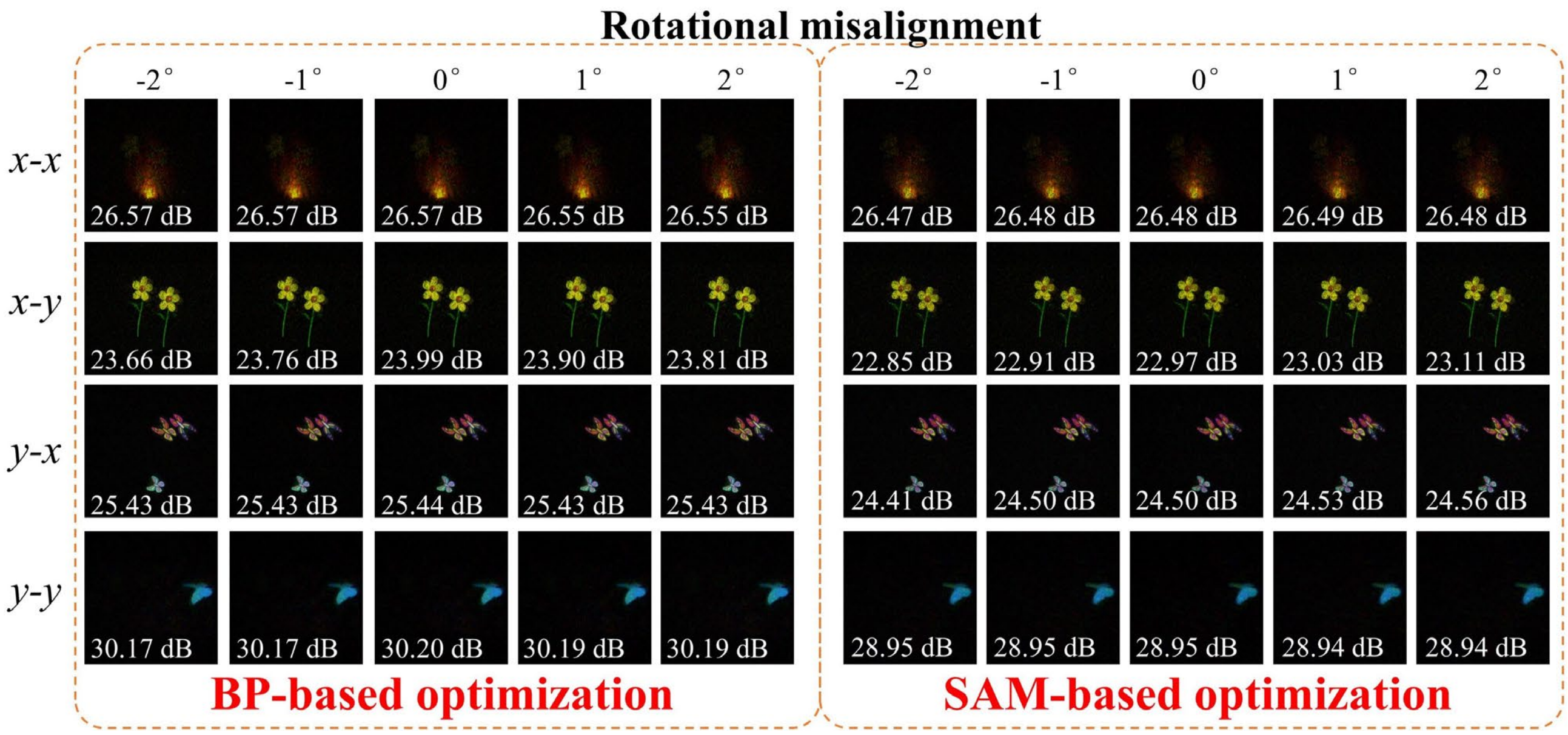

Figure S23. Effect of rotational misalignment in the secondary metasurface on 288-channel holographic performance, comparing results obtained with BP- and SAM-based optimization.

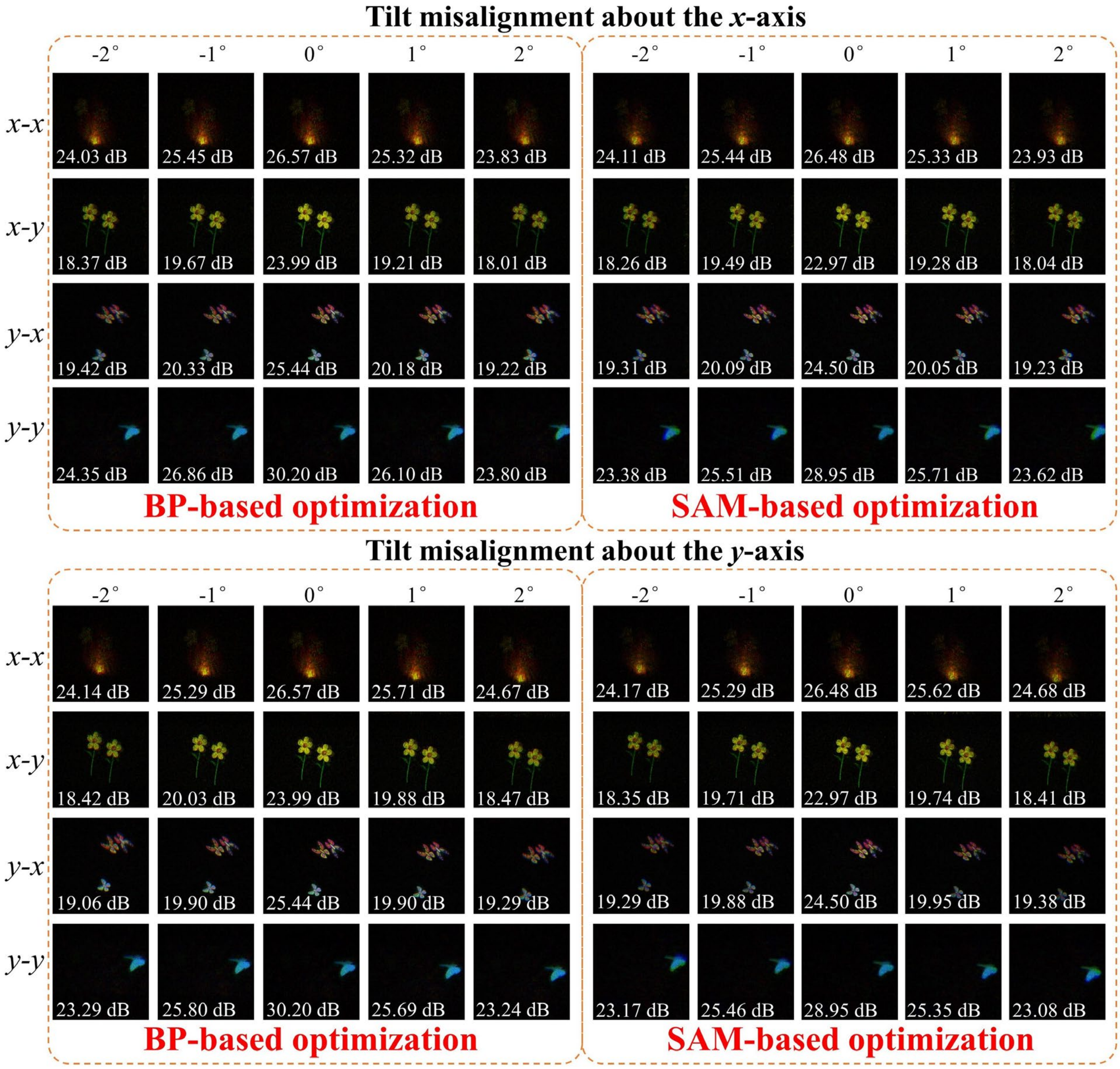

Figure S24. Impact of mutual tilt misalignment between bilayer metasurfaces on 288-channel holographic performance using BP- and SAM-based optimization, respectively.

References

[1] P. Foret, A. Kleiner, H. Mobahi, "Sharpness-aware minimization for efficient machine learning," arXiv:2010.01412, **2021**.

[2] Z. Yao, A. Gholami, K. Keutzer, M. W. Mahoney, "PyHessian: Neural networks through the lens of the Hessian," in *2020 IEEE International Conference on Big Data (Big Data)*, IEEE, Atlanta, GA, USA, **2020**, pp. 581–590.
